# A consensus mathematical model of vaccine-induced antibody dynamics for multiple vaccine platforms and pathogens

**DOI:** 10.3389/fimmu.2025.1596518

**Published:** 2025-06-26

**Authors:** Kristen M. Wilding, Carmen Molina-París, Jessica Z. Kubicek-Sutherland, Benjamin McMahon, Alan S. Perelson, Ruy M. Ribeiro

**Affiliations:** ^1^ Theoretical Biology and Biophysics Group, Los Alamos National Laboratory, Los Alamos, NM, United States; ^2^ Physical Chemistry and Applied Spectroscopy Group, Los Alamos National Laboratory, Los Alamos, NM, United States

**Keywords:** vaccine, antibody dynamics, B cell, memory, vaccine platform, SARS-CoV-2, ebola, mathematical model

## Abstract

**Introduction:**

Vaccine platforms used in successful, licensed vaccines have varied among pathogens. However, antibody level is still the main clinical correlate of protection in most approved vaccines. Decisions as to the best vaccine platform to pursue for a given pathogen may be informed through improved understanding of the process of antibody generation and its temporal dynamics, as well as the relationship between these processes and the type of vaccine.

**Methods:**

We have analyzed the dynamics of antibody generation for different vaccine platforms against diverse pathogens, and developed a consensus mathematical model that captures antibody dynamics across these diverse systems. Initially, the model was fitted to a rich dataset of antibody and immune cell concentrations in a SARS-CoV-2 vaccine experiment. We then used concepts from machine learning, such as transfer learning, to apply the same model to a variety of systems, involving different pathogens, vaccine platforms, and booster dose use/timing, fixing most parameter values relating to the dynamics of the immune system.

**Results:**

The model includes B cell proliferation and differentiation, as well as the generation of plasma cells, which secrete large amounts of antibody, and memory B cells. Overall, the model describes antibody generation in all systems tested well and shows that the main differences across platforms are related to the dynamics of antigen presentation.

**Discussion:**

This model can be used to predict antibody generation in pairs of vaccine platform/pathogen, allowing for the use of in silico results to narrow down experimental burden in vaccine development.

## 1 Introduction

Over the past two decades multiple infectious diseases have emerged causing outbreaks which resulted in thousands to tens of millions of deaths worldwide (1). It is expected that these disease emergences will become increasingly more frequent with human activities impacting on climate, and in turn, wild ecosystems, habitats, and biodiversity (1). Vaccines are a critical tool against epidemics and pandemics, offering protection from clinical or severe disease, and reducing pathogen transmission (2–4). Approved vaccines span multiple platforms such as live attenuated, protein subunit, mRNA, and viral vector vaccines, with the most successful platform varying depending on the pathogen (5). For example, mRNA vaccines, adenovirus vectored vaccines, and protein subunit vaccines have been widely used to mitigate infection with SARS-CoV-2 (6–10); in contrast, the leading Ebola vaccine uses a vesicular stomatitis virus (VSV) vector (5, 11). In the face of an emerging pathogen, decision support is needed to suggest which platforms hold the most promise for a particular pathogen. While this is a complex, multi-faceted problem, mechanistic modeling of the adaptive immune response can provide valuable quantitative insights into the underlying processes of both humoral and cellular immune responses (12–14), and in turn, inform selection of best vaccine candidates for a given pathogen.

Mechanistic immune dynamics models have been used successfully to study infections such as HIV (15–27), hepatitis B virus (28–35), SARS-CoV-2 (12, 36–42), and others. Similar models have also been used to evaluate the immune responses to vaccination (43, 44). Antibody titers are often considered the strongest correlate of vaccine-induced protection (5), and are thus, frequently, an important variable in such dynamical models. However, these models are usually designed and fitted specifically to a single pathogen and vaccine platform, requiring rich longitudinal datasets for reliable and accurate parameter estimation. To better enable analysis of antibody dynamics on the small datasets more likely to be available early in vaccine studies, we sought to develop a consensus model that could be used both for multiple pathogens and different platforms without the need to re-fit all model parameters. Recently, Xu et al. proposed a single model structure to capture the dynamics of the immune response to SARS-CoV-2 vaccines from two different platforms (Adenovirus and mRNA) (43). However, the model used very limited data (just 4 to 5 data points for each case), and did not specifically consider the generation of long-lived cellular species, such as memory B and long-lived plasma cells, which are crucial for the longevity of the humoral immune response (45). Furthermore, the previous work did not evaluate how the model would extend to vaccine responses for pathogens beyond SARS-CoV-2.

Here, we develop and fit a consensus mathematical model to antibody titer and memory B-cell data, when available, from multiple vaccine platforms for two different pathogens: SARS-CoV-2 and Ebola virus. We present a model structure that explicitly considers identifiable cell groups contributing to lasting immunity: memory B and long-lived plasma cells. We fit the model initially to a rich dataset from an adenovirus vectored SARS-CoV-2 vaccine study in rhesus macaques that contains longitudinal measurements of both IgG titers and IgG^+^ memory B cell frequency (46). To enhance the generalizability of the model, we also simultaneously fit the model to longitudinal IgG titers from an adenovirus vector Ebola vaccine study in cynomolgus macaques (47). A third dataset, a SARS-CoV-2 mRNA vaccine study in humans with longitudinal measurements for both receptor binding site (RBD) specific IgG and IgG^+^ memory cells (8), serves as a validation set in selecting the optimal consensus model. We show that inclusion of both long-lived plasma cells and antibody-dependent clearance of antigen improves the model fits, with biologically relevant mechanisms, allowing the model to more closely fit aspects of long-term longitudinal Ab titers - such as biphasic Ab declines and inter-individual variability in responses to booster doses. Finally, we apply the model to six additional datasets from various SARS-CoV-2 or Ebola virus vaccine platforms. We show that our consensus model is able to successfully capture the IgG dynamics of each dataset by re-fitting only a small subset of parameters related to antigen dynamics and long-lived plasma cell dynamics, keeping core immune dynamics parameters fixed. In addition to enabling the model to be applied to more sparse datasets, the ability to fix core immune dynamics parameters suggests a degree of similarity in the underlying processes (e.g. cell transition rates) involved in the immune response to vaccination and suggests that variability in antigen presentation may be critical for differences in the early immune response generated by different vaccines, platforms, or pathogens.

## 2 Methods

### 2.1 Data

The datasets analyzed in this study can be grouped into data used to develop the core of the consensus model and data to which the core consensus model was applied. All datasets were obtained from the literature, using the data files published with the reference where applicable, or digitized from the reference using Plot Digitizer (48). The datasets are described in **Table 1**. Additional information about the fitted vaccine datasets is contained in **Supplementary Table S4**.

**Table 1:** Literature datasets used.

Use	Pathogen	Vaccine platform	Data type	Species	Ref.
Platform dynamics	SIV	Ad vector	mRNA	C57BL/6 mice	(49)
	GFP	mRNA	mRNA/GFP positivity	cynomolgus macaques	(50)
	Ebola	VSV vector	mRNA	humans	(51)
Construction	SARS-CoV-2	Ad vector	IgG titer, IgG^+^ memory B	rhesus macaques	(46)
	EBOV	Ad vector	IgG titer	cynomolgus macaques	(47)
Validation	SARS-CoV-2	mRNA	IgG concentration, IgG^+^ Memory B	humans	(8)
Application	SARS-CoV-2	mRNA	IgG titer	humans	(52)
	SARS-CoV-2	protein subunit	IgG titer	rhesus macaques	(53)
	SARS-CoV-2	protein subunit	IgG titer	rhesus macaques	(54)
	EBOV	VSV vector	IgG titer	cynomolgus macaques	(55)
	EBOV	protein subunit	IgG titer	humans	(56)
	EBOV	protein subunit	IgG titer	C57BL/6 mice	(57)

### 2.2 Mathematical models

We propose a simplified model of B cell activation by vaccine antigen, followed by proliferation, affinity maturation, and differentiation into plasma and memory cells. We assume that antigen stimulates a set of B cells, which can belong to different clones. The dynamics of these B cells, and the plasma cells and memory cells they generate are given by Equations 1–7 below


(1) $$\frac{dB_{1}}{dt}=\rho_{1}\frac{Ag}{K+Ag}B_{1}-\psi B_{1}-\delta_{B}B_{1}\;$$

(2) $$\frac{dB_{2\ldots 7}}{dt}=\rho_{1}\frac{Ag}{K+Ag}B_{n}+\psi B_{n-1}-\psi B_{n}-\delta_{B}B_{n}\;\;$$

(3) $$\frac{dB_{8}}{dt}=\rho_{2}\frac{Ag}{K+Ag}B_{8}+\psi B_{7}-\left(\mu +\pi +\delta_{B}\right)B_{8}\;\;$$

(4) $$\frac{dP}{dt}=\pi B_{8}+\pi_{2}M^{*}-\left(\delta_{P}+\lambda \right)P\;\;$$

(5) $$\frac{P_{L}}{dt}=\lambda P-\delta_{L}P_{L}\;\;$$

(6) $$\frac{dM}{dt}=\mu B_{8}+\mu_{2}M^{*}+\left(\rho_{M}-\delta_{M}\right)M-\phi_{M}\frac{Ag}{\frac{K}{m_{M}}+Ag}M\;\;$$

(7) $$\frac{dM^{*}}{dt}=\phi_{M}\frac{Ag}{\frac{K}{m_{M}}+Ag}M+\rho_{2}\frac{Ag}{\frac{K}{m_{M}}+Ag}M^{*}-\left(\mu_{2}+\pi_{2}+\delta_{B}\right)M^{*}\;\;$$

Here, *Ag* is the concentration of protein antigen (see below), which recruits naive B cells into the response and induces B cell proliferation at saturating rate *ρ*_1_, with half-maximal saturation constant *K*. The germinal center (GC) reaction is represented simply by a progression (differentiation) of B cells through different stages *B*_2_ to *B*_8_ at rate *ψ*, where they experience antigen-dependent proliferation (required by affinity maturation), and at each stage they can be lost at per capita rate *δ_B_*. Equation 3 represents *B*_8_ cells that have undergone affinity maturation (including somatic hypermutation) and isotype switched to IgG^+^ B cells and can exit the germinal center reaction. At this stage, we allow the B cell proliferation rate of *B*_8_ cells to increase to *ρ*_2_, in accordance with reports that mature B cells (and memory cells) proliferate faster than naive (58). These cells can differentiate into plasma cells, *P*, at rate *π*, or into memory cells, *M*, at rate *µ*. Plasma cells, *P*, may also be generated by differentiation from activated memory cells, *M* ^∗^, at rate *π*_2_. P can further differentiate into long-lived plasma cells, *P_L_*, at per capita rate *λ*, or die at per capita rate *δ_P_*, whereas *P_L_* are lost at a lower per capita rate, *δ_L_*. An alternative fate of cells exiting the germinal center reaction is differentiation into resting memory cells, *M*, which proliferate and die at per capita rates *ρ_M_* and *δ_M_*, respectively. These cells can also be recruited into a secondary response by antigen at saturating rate *φ_M_*, with half-maximal saturation constant *K/m_M_*. Memory cells activate more readily than B cells in the primary response (58), thus their half-maximal saturation constant is smaller by a factor *m_M_*. Memory cells activated in a recall response, *M* ^∗^, proliferate at saturating rate *ρ*_2_, differentiate into plasma cells at rate *π*_2_, and can go back to long-lived memory cells at rate *µ*_2_ or die at rate *δ_B_*. Antibodies, *A*, can be produced by plasma cells at per capita rate *θ_P_*, by long-lived plasma cells at per capita rate *θ_L_* and by mature IgG^+^ B cells after undergoing the germinal center reaction (*B*_8_ cells) at per capita rate *θ_B_*, although the latter contribute much less, and we assume a 10^2^-fold reduced secretion rate compared to plasma cells (59). In turn, antibodies are cleared from circulation at rate *δ_A_*, and thus, antibody dynamics is given by


(8) $$\frac{dA}{dt}=\theta_{P}P+\theta_{B}B_{8}+\theta_{L}P_{L}-\delta_{A}A\;$$

We note that in some experiments measurements of B cell populations are expressed as frequencies out of total B cell numbers. If *T* represents the total concentration of B cells, which we assume to be approximately constant during a vaccine immune response, as the majority of B cells are not antigen specific, we can divide every equation (Equations 1–7) by *T*, without changing any term or any parameter. Thus, those equations are invariant whether we are referring to cell concentrations (mL^−1^) or frequencies. Equation 8 would also remain the same, but the interpretation of the antibody production rates *θ_x_* where the subscript *x* could be *P*, *B*, or *L*, would be slightly different. We should also note that we evaluated many different alternatives in developing the model presented above, including different forms of the antigen-driven activation of B cells, how many B cell stages to include, whether to have long-lived plasma cells differentiate from plasma cells or directly from mature B cells, whether we should differentiate between memory cells (*M*) and activated memory cells (*M* ^∗^), or if memory cells should become activated B cells (*B*_8_) when encountering antigen, and other choices. We do not present all of these alternatives here, but a subset of them is presented in section 3.1. We also evaluated models that included CD4^+^ T cell help, an important process for efficient B cell memory formation (55, 60), however we found we did not have enough data to constrain their this mechanisms of action, as we found few datasets in the literature with longitudinal measurements of antigen-specific CD4^+^ T cells, B cells, and Ab levels following vaccination. CD4^+^ T cell help drives germinal center formation and influences the proliferation rates of B cells in the primary, and possibly the secondary, response to antigen (61–64). Without explicit inclusion of CD4^+^ T cell help in the model, we anticipate that the effect of T cell help will be absorbed into the values of the different parameters, such as *K* or *µ*, which increase the B cell and memory B cell expansion rates. While interactions with CD4^+^ T cells may also influence the relative levels of plasma cells versus memory B cells, the extent and mechanism of this dependence are not well understood (61, 64), and therefore, in light of the data limitations discussed, we assumed the relative rates of plasma cell and memory cell generation are approximately constant.

As described above, the B cell response is induced by antigen, and we are particularly interested in vaccine antigens. Different vaccine platforms (*e.g.*, vectored vaccines, protein subunit vaccines, DNA, or mRNA vaccines) may lead to different dynamics of the antigen, *Ag*, in the equations above. Therefore, we need to account for these different possibilities, without including undue complexity, for which we do not have data for parameterization. Thus, we assume that either the antigen is delivered directly into the system, as is the case with subunit vaccines, or that genetic material, *R*, coding for the antigen is delivered, for example with vectored vaccines (such as adenovirus-based vaccines) or with standard mRNA vaccines. Further, the genetic material may be delivered in a replication-competent form (*e.g.*, vesicular stomatis virus vectored vaccines or self-amplifying RNA) or not (*e.g.*, adenovirus vectored vaccines or mRNA vaccines). We also assume for simplicity that different modalities of antigen presentation (*e.g.*, with antigen presenting cells, such as follicular dendritic cells (FDCs), free protein or others) are averaged together into a single species, *Ag*, representing all antigen seen by B cells. Antigen dynamics is described by the equation


(9) $$\frac{dAg}{dt}=k_{t}\frac{R}{K_{R}+R}-\delta_{Ag}Ag-k_{b}AgA\;$$

where the term *k_t_R/*(*K_R_* + *R*) represents antigen production from the genetic template, *R*, using a saturation term with half-maximal constant *K_R_*. Antigen can be cleared by antibody-independent processes at rate *δ_Ag_* and is also cleared by forming antibody-antigen complexes at rate *k_b_*. As these complexes are assumed to be cleared we do not consider them further. In the case of vaccine modalities where the antigen is injected directly into the system, *k_t_* = 0 and the bolus of antigen is simply cleared from circulation. The genetic template of for antigens produced by the host has dynamics governed by


(10) $$R\left(t\right)=\{\begin{array}{ll} R_{0}e^{rt}\;, & t<T_{off}\;\\ R_{0}e^{rT_{off}}e^{-\delta_{R}\left(t-T_{off}\right)}\;, & T_{off}\leq t\; \end{array}$$

where *R*_0_ is the initial amount of the genetic template introduced by vaccination, *r* is the replication rate of the genetic material, *T_off_* is the end of the period during which the process of replication is active, and *δ_R_* is the clearance rate of the genetic material. For non-replicating cases, such as mRNA vaccines, *r* = 0 and *T_off_* = 0. The exponential form is chosen to mirror the exponential-like growth observed for many viruses (65–68), since these replicative vaccines are based on viral vectors.

Some vaccination protocols include multiple vaccine boosters. We account for this in our antigen dynamics, by either adding a bolus of antigen at appropriate times, when this is administered directly (such as with subunit vaccines), or by accounting for these boosters in a modified *R* equation given by


(11) $$R\left(t\right)=\{\begin{array}{ll} R_{0}e^{rt} & t<T_{off}\;,\\ R_{0}e^{rT_{off}}e^{-\delta_{R}\left(t-T_{off}\right)} & T_{off}\leq t<T_{boost},\\ R_{0}e^{rT_{off}}e^{-\delta_{R}\left(t-T_{off}\right)}+R_{02}e^{r(t-T_{boost})} & T_{off}\leq t<T_{boost}+T_{off2}\\ \begin{array}{l} R_{0}e^{rT_{off}}e^{-\delta_{R}\left(t-T_{off}\right)}+\;R_{02}e^{rT_{off2}}\\ e^{-\delta_{R}(t-(T_{boost}+T_{off2}))} \end{array} & (T_{{\;}_{boost}}+T_{off2})\leq t \end{array}$$

where *T*_boost_ and *T_off2_* have straightforward meaning. For further boosters, this idea is replicated.

### 2.3 Model fitting to data and parameter estimation

To fit the model to the data, we used a non-linear mixed-effect modeling approach with the software Monolix version 2024R1 (Lixoft SA, Antony, France) (69). We either fit the model to antibody data or simultaneously to antibody and memory B cell frequencies, when available (more details below). We modeled the measured antibody levels (*y*) in a log_10_ scale and the frequencies of B cells (*z*) on an linear scale, for individual *i* at time *j* as $y_{ij}={\text{log}}_{10}A\left(t_{j}\right)+\sigma_{A}$ and $z_{ij}=\left(B_{8}+M+M^{*}\right)+\sigma_{B}$ , respectively, with $\sigma_{A}∼N\left(0,\sigma_{A}^{2}\right)$ and $\sigma_{B}∼N\left(0,\sigma_{B}^{2}\right)$ , the error for the logged antibody levels and B cell frequencies, respectively (Note that IgG^+^ “memory B cells” reported in He et al. (46) were gated based on CD27 expression, which is also expressed by cells exiting the germinal center (70, 71), and thus, we fit the sum *B*_8_ + *M* + *M* ^∗^, which represent frequencies as described above. Though plasma cells are also often CD27 positive, He et al. gated plasma cells out via absence of CD20, so they were not included in the summation.) In the mixed-effect approach, we assume that a model parameter, *η_i_*, is drawn from a distribution with a fixed part *θ*, which is the value of the parameter in the population, and a random term *φ_i_*, which is assumed to be normally distributed with zero mean and standard deviation *σ_θ_*. Typically, we assume that the parameters follow a lognormal distribution, which ensures their positivity. Model fitting allows estimation of the population parameters and the variances of the distribution of each parameter. We fixed some model parameters at literature values (see **Tables 2**, **3**), because this reduces the number of parameters and makes it easier to determine the remaining parameters (e.g., more consistent across individuals and scenarios). The initial value of the first B cell stage, *B*_0_, is fitted for each dataset, and values of *R*_0_ or *Ag*_0_ are fixed according to the antigen template or protein antigen dosages reported with the datasets. We fix *R*_0_ and *Ag*_0_, because *R*_0_ (*Ag*_0_) trade-off against *K_R_* (*K*) in the term $\frac{R}{K_{R}+R}$ in Equation 9 $\frac{Ag}{K+Ag}$ in Equation 1), and so these initial conditions can not be well determined. Fixing these values allows the model to differentiate between individuals with different doses (at least when fitting them simultaneously) and also estimate the parameters *K_R_* and *K*. We anticipate that bio-availability may differ between vaccine platforms, however, this effect is again largely absorbed into the *K_R_* or *K* parameters. All other initial values are set to 0. For the He et al. (46) and Goel et al. (8) datasets, both IgG^+^ antibody titers and memory B cell frequencies are available and we fitted them simultaneously. For the other datasets, we fitted the antibody titers only (see **Table 1**).

**Table 2:** Description of model parameters.

Parameter	Description	Fitting
$R_{0},\;R_{02},\ldots$	Antigen template dose	From study
$Ag_{0},\;Ag_{02},\ldots$	Protein antigen dose	From study
$T_{boost},T_{boost2}$	Time of booster dose	From study
*T_off_*, *T_off2_*	Duration of template replication	From template dynamics fits
*r*	Template replication rate	From template dynamics fits
*δ_R_*	Template decay rate	From template dynamics fits
*ρ*_1_	Max proliferation rate of activated B cells	Fixed
*ρ*_2_	Max proliferation rate of GC-experienced B cells	Fixed
*δ_P_*	Death rate of short-lived plasma cells	Fixed
*δ_A_*	Decay rate of IgG Ab	Fixed
*π*	Differentiation rate to PCs	Core
*µ*	Differentiation rate to resting memory, *M*	Core
*δ_B_*	Death rate of activated B cells	Core
*θ_P_*, *θ_L_*	Ab production rate from *P*, *P_L_*	Core*
*ψ*	Transition rate between B cell stages	Core
*ρ_M_* - *δ_M_*	Net turnover of resting memory B cells	Core
$\phi_{M}$	Resting memory B cell activation rate	Core
*π*_2_	PC formation from activated memory	Core
*µ*_2_	Memory cell formation from activated memory cells	Core
$m_{M}$	Memory cell half-maximal activation reduction factor	Core
*k_t_*	Translation rate	Core
*λ*	Differentiation rate of *P* to *P_L_*	Fitted**
*δ_L_*	Decay rate of *P_L_*	Fitted**
*B*_0_	Initial reactive B cell frequency	Fitted
*K_R_*	Template concentration for half-maximal translation	Fitted
*δ_Ag_*	Protein antigen decay rate	Fitted
*k_b_*	Ab-Ag complex formation rate	Fitted
*K*	Ag concentration for half-maximal proliferation	Fitted

“From study” parameters are taken from the journal article reporting each dataset (see **Supplementary Table S4**).

“From template dynamics fits” are fixed at the values in **Supplementary Table S1** from the corresponding platform.

“Core” parameters are fitted initially on the “construction” datasets and fixed for all remaining fits.

*Re-fitted for special cases as specified in the text (*i.e.*, different Ab and memory B cell units). **Re-fitted when follow-up is long enough to capture the second phase of antibody decay.

**Table 3:** Fixed and core fitted model parameters.

Parameter	Value [s.e.]	Unit	Reference
*ρ*_1_	2.5	d^−1^	(58, 90)
*ρ*_2_	4.0	d^−1^	(58, 60, 91, 92)
*δ_P_*	0.35	d^−1^	(93)
*δ_A_*	*NHP:* 0.050	d^−1^	(94)
	*human:* 0.033	d^−1^	(95)
	*mouse:* 0.087	d^−1^	(96)
*π*	0.023 [0.017]	d^−1^	
*µ*	0.050 [0.015]	d^−1^	
*δ_B_*	0.14 [0.045]	d^−1^	
*θ_P_*, *θ_L_*	1.2 ×10^7^ [5.6 ×10^6^]	EU/day	
*ψ*	0.27 [0.07]	d^−1^	
*ρ_M_* - *δ_M_*	-0.011 [0.0014]	d^−1^	
$\phi_{M}$	0.95 [0.11]	d^−1^	
*π*_2_	1.8 [0.4]	d^−1^	
*µ*_2_	4.1 [1.0]	d^−1^	
$m_{M}$	3237 [2.1]	unitless	
*k_t_*	3255 [13.8]	[Ag]/d	
*λ*	*SARS-CoV-2:* 0.0058 [0.0013]	d^−1^	
	*Ebola:* 0.0049 [0.0015]	d^−1^	
*δ_L_*	*SARS-CoV-2:* 0.0032	d^−1^	
	*Ebola:* 0.000038	d^−1^	

Monolix maximizes the likelihood using the Stochastic Approximation Expectation Algorithm (SAEM), which is very efficient for a wide range of models (69). As with other algorithms for non linear models, the user provides initial guesses for the parameters. To avoid solutions which could be sub-optimal due to the starting guesses, in each case, we run the estimation starting from more than twenty different initial parameter guesses (making use of the automated assessment feature in Monolix). On a Mac laptop (M1 Max chip, 32GB memory), assessments of 20 initial guesses took between 30 min and 5 hours, depending on the number of parameters fitted and the size of the dataset. From all these, the fit with the lowest log-likelihood was selected for each dataset.

To constrain our model better, when possible (*i.e.*, if data were available), we fitted the sub-model of antigen dynamics (Equations 9–11) to data of specific vaccine platforms. To this end, we used data on SIV mRNA expression in the draining lymph node from an adenoviral vector vaccine study (49), data from VSV RNA in blood after an Ebola vaccine using the VSV platform (51), and both mRNA levels and GFP positivity (protein levels), fitted simultaneously, for an mRNA vaccine study (50). Thus, for these three platforms, we fitted that part of the model first, and fixed the corresponding parameters when fitting the antibody data using the other equations. Note that these antigen dynamics parameters were fixed to the estimated ones, even if the mRNA in those studies was not the same as in the vaccine of interest. For example, we used adenoviral mRNA expression of a SIV vaccine antigen as a proxy for mRNA expression of SARS-CoV-2 spike in adenoviral vaccines.

### 2.4 Parameter sensitivity

Sensitivity of various cell- and antibody- dynamic metrics (*e.g.*, peak magnitude, peak times and time to decay to 50% of peak magnitude) to model parameters was assessed in R (version 4.3.2). We performed 5 × 10^4^ simulations with random variations of model parameters within the distributions estimated in the maximum likelihood fit of the He et al. (46) data. We evaluated correlations between these metrics and the simulation parameters using the Hmisc package (72) to calculate correlations and the corrplot package (73) to visualize the correlations. We also performed random forest regression using the randomForest package (74) to predict antibody peak level (“Apeak.mag”) and the time to decay to 50% of the peak antibody level (“Apeak50.time”) from the parameters of the simulation. The importance of each parameter for a given mathematical model was determined using the caret package in R (75).

### 2.5 Statistical analysis

We also compared model parameters using R package Hmisc package (72) to perform a Student t test to assess significance in differences between the means of individual-level parameters estimated by Monolix. Calculated p-values were adjusted using a Bonferroni multiple test correction.

## 3 Results

### 3.1 Determining model structure

Our model structure (Equations 1–11) is built with two specific aims in mind (1): making the model immunologically relevant with measurable cell populations, and (2) using a generalizable structure to bridge vaccine platforms and antigens in an interpretable way. In pursuit of these aims, we evaluated various model structures to determine a robust ODE system which would capture the relevant processes involved in the humoral immune response in an interpretable and transferable manner. To develop the model presented in section 2, we considered both immunological relevance and fit quality, as determined by the Corrected Bayesian Information Criteria (BICc) in Monolix (76).

Our model structure captures the basic progression of B cells from activation to antibody production and memory cell formation. Upon binding a cognate antigen, naive B cells begin to proliferate. These activated, proliferating B cells are generally thought to differentiate towards one of two primary fates: plasma cells or memory B cells. Plasma cells produce large amounts of antibody and are generally short-lived. However, a small subset of plasma cells become long-lived plasma cells (*P_L_*), which can persist for decades and are critical for long-term immune protection (77). Still, we initially considered a model without long-lived plasma cells, but preliminary fits indicated that the model was unable to capture the two-phase decay observed in the long-term dynamics of antibody titers (see **Supplementary Table S2** and supplementary information). This is corroborated by previous work which has directly linked the two-phase decay of antibody titers to the half-life of short-lived and long-lived plasma cells (78, 79), suggesting the need for both cell types to capture longitudinal antibody dynamics. Differentiation into *P_L_* has been suggested to occur from activated memory B cells (80), or as a function of B cell receptor (BCR) affinity (81, 82), though recent studies suggest they may develop from short-lived plasma cells which are able to migrate to a niche supporting long-term survival (81, 83–85). Therefore, in our model, we assume that activated B cells may differentiate into short-lived plasma cells or memory cells, and that a fraction of short-lived plasma cells (PCs) then go on to persist as *P_L_*. We assume that *P_L_* s produce antibody at the same rate as *P*s (*θ_P_* = *θ_L_*), as without data on the relative levels of *P_L_* and *P* cells, the relationship between *θ_P_* and *θ_L_* would be difficult to constrain.

Memory B cells can be further divided into activated memory cells (*M* ^∗^), which rapidly proliferate and differentiate into plasma cells (58), and resting memory cells, *M*, which persist in the body for long periods of time, ready to re-activate and initiate a recall immune response upon re-exposure to antigen. Although, we initially tried models with only one population of memory cells, we found that models with two populations (*M* and *M* ^∗^) performed better, in part because the activated memory cells can re-seed the resting memory compartment. Thus, we include them separately in our model accounting also for differences in proliferation rates and differentiation potential. In addition, both types of cells were measured in the He et al. data analyzed in this study (46).

Another important consideration for our model is to accurately capture the timing of affinity maturation and antibody class-switching. The antibody response generally begins with IgM. As the immune response progresses, B cells undergo class-switching to produce IgG or IgA antibodies, most commonly in the context of a germinal center reaction. IgG antibodies typically have longer half-lives than other classes, such as IgM or IgA, and are the most frequent correlate of vaccine-induced protection (5). In the study by He et al. (46), IgG^+^ memory B cells were gated by their expression of CD27, which may also be expressed on germinal center B cells (70, 71). Therefore, we wanted to include two separate classes of B cells in our model to represent B cells before and after class-switching in the GC. Class-switching typically takes between 15–21 days after vaccination (60), and we evaluated a version of the model which employs the “linear chain trick” to narrow the distribution of “switch times” for the B cells and avoid prediction of class-switched cells too early. This approach is a variation of the well recognized method in ODE models, both for within host models (65, 66, 86, 87) and epidemiology models (88), in which adding transitional “stages” to an ODE model narrows the distribution of residence times as the number of “stages” increases, generating an Erlang distribution rather than the exponential distribution characteristic of traditional first-order kinetics (65, 66, 88). We tried models with 2, 5, 8, 10, and 15 B cell “stages,” and found that an 8 stage model was appropriate to generate good fits. Relative quality of fits for the models with 2 stages and 8 stages are shown in **Supplementary Table S2**. Other transitions in the model are left as exponential type transitions, as is commonly used in within-host models, for simplicity and because we do not have data to constrain the additional parameter required for alternative transition types, such as the Erlang distribution previously discussed (65, 66, 88).

Finally, we also experimented with different model structures for the antigen dynamics. From a simple exponential increase followed by a decrease for replicating antigens, or just an exponential decrease for non-replicating antigens, to the more detailed final model in Equations 9 and 11. In addition, we also analyzed the effect of existing antibody on the immune response after vaccination. Antibody may reduce the available vaccine antigen by binding to it and accelerating its clearance (7, 89). Thus, we evaluated whether inclusion of a reaction between existing Ab and the vaccine Ag would enhance the model’s fit to the data. We found that adding an Ab-Ag binding/clearance term markedly improved the fit of the model (**Supplementary Table S2**). This is consistent with experimental results which have found that the fold-increase of antibody titers after boosting is inversely correlated with pre-boost antibody titers (7). The addition of the Ab-Ag binding/clearance term therefore improves the model by providing biologically-relevant flexibility in fitting the variability of post-boost Ab titer increases.

**Figure 1:**
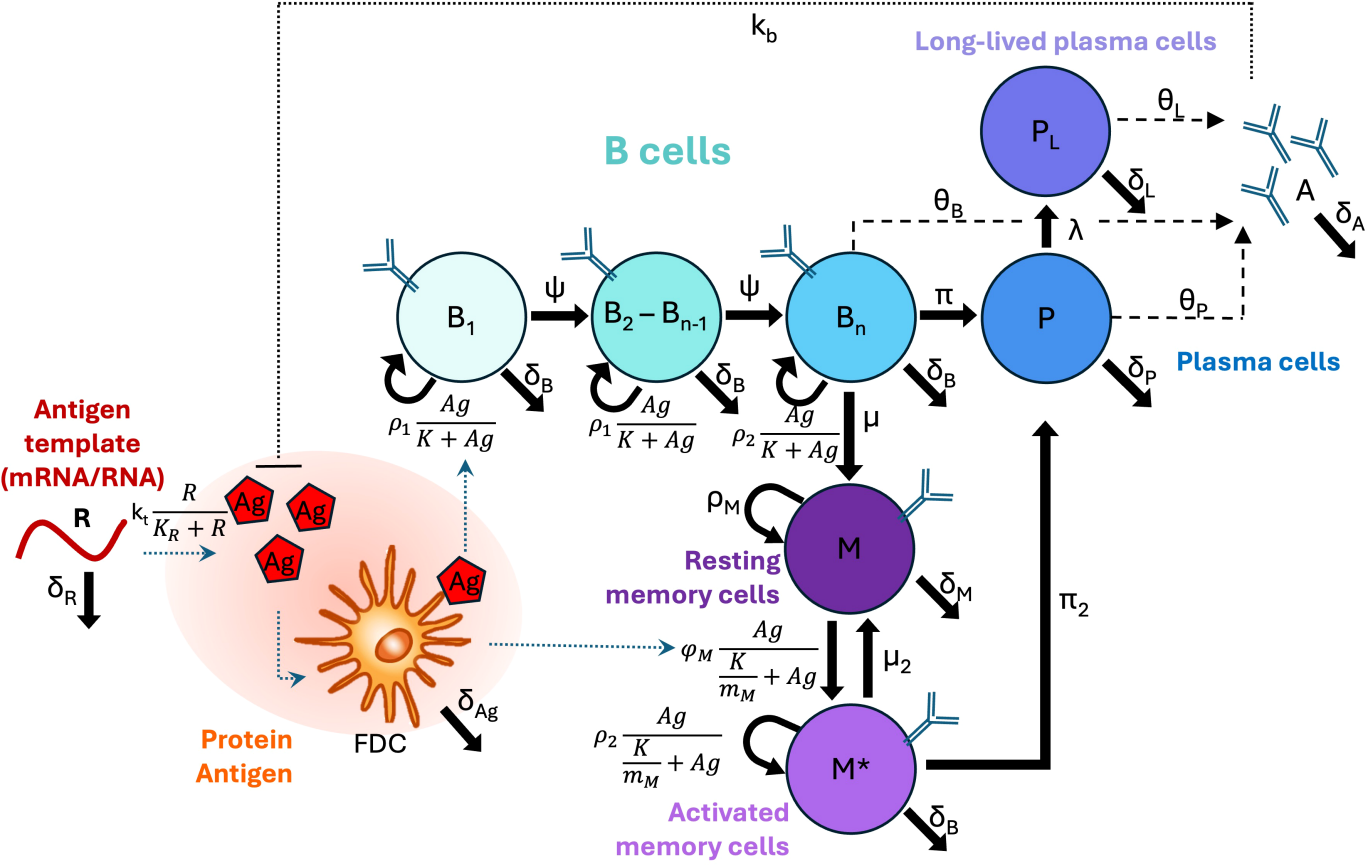
Model schematic. B cell types are shown as circles, and differentiation, proliferation, or death rates are indicated by solid arrows. Secretion is indicated by dashed arrows. Other types of interactions (*e*.*g*., inhibition, uptake, or presentation) are indicated by dotted lines. Note that in our implemented model *B*_n_=*B*_8_.

Consideration of these different biological factors and statistical comparison of the different model structures led us to our final model presented in detail in the Methods section, and which we use for the remainder of our analysis. A model schematic is shown in **Figure 1**.

### 3.2 Parameter estimation and model validation

We constructed our model primarily using a rich dataset of longitudinal SARS-CoV-2 receptor binding domain (RBD)-specific IgG titers and IgG^+^ memory B cells after an adenovirus vaccination protocol in rhesus macaques (see He et al. (46)). This dataset contains frequent measurements of those two quantities for up to 343 days after a primary vaccine dose, including two time points after a booster was given. We only included the post-boost data from macaques that received a homologous booster dose. An adenovirus-based vaccine delivers the DNA template of the antigen (here RBD), which is transcribed into mRNA and then translated into the antigen protein. Thus, to further constrain our model, we first independently estimated the dynamics of mRNA transcripts coding for the vaccine antigen (**Supplementary Figures S1**–**S3**; **Supplementary Table S1**). This was done by fitting the growth and decay rates in Equation 10 to measurements of mRNA transcripts in the draining lymph node previously reported for several adenovirus vectors (49). We then assumed that mRNA dynamics is approximately independent of the type of DNA delivered by the adenovirus vector (*i.e.*, whether it codes for SIV or SARS-CoV-2) and fix these estimated parameters (shown in **Supplementary Table S1**) while fitting the antibody and memory B cell dynamics model to the He et al. dataset (46).

**Figure 2:**
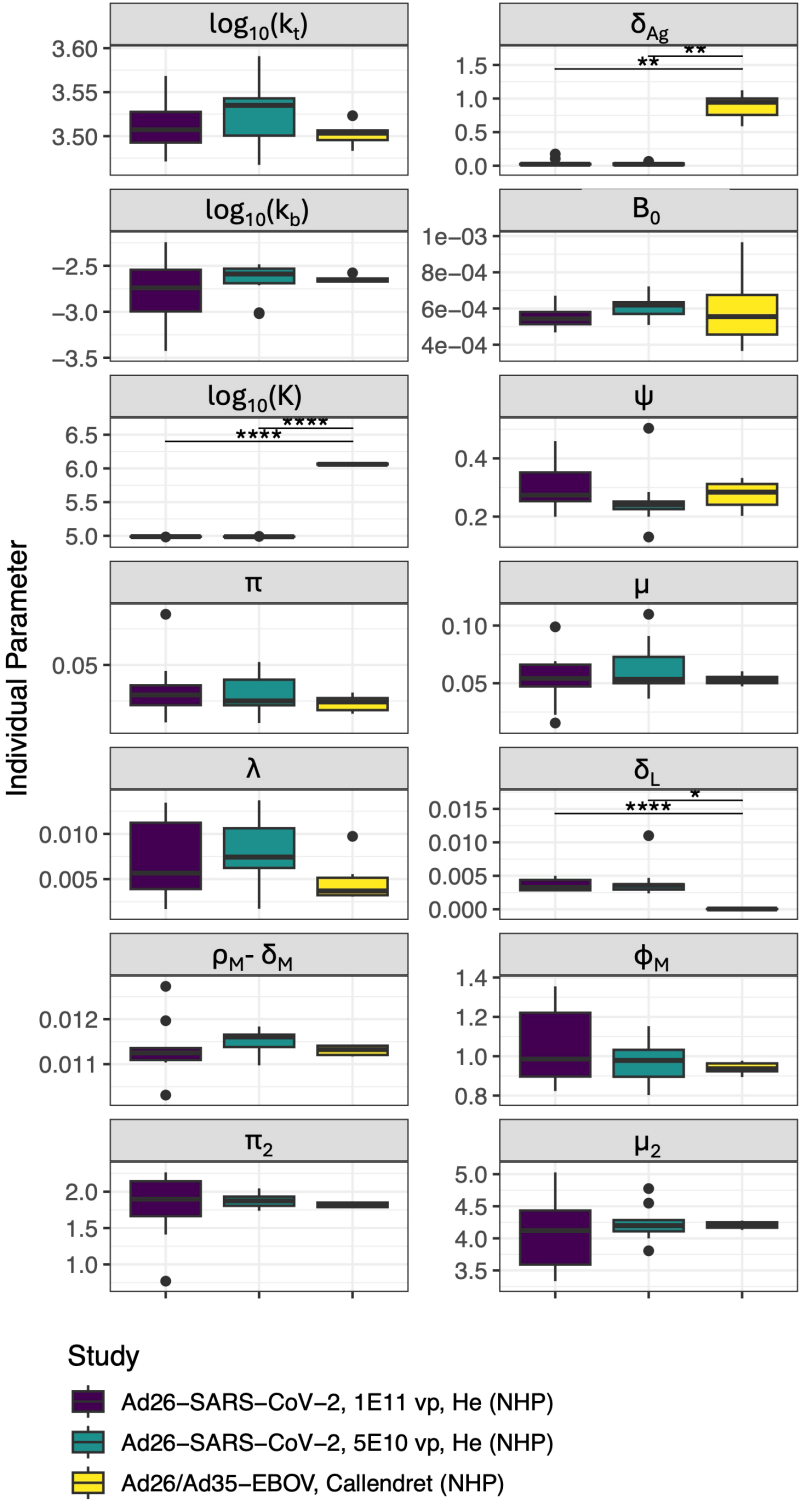
Distribution of parameters for individual NHPs in each study/dose group (*δ_B_*, *δ_A_*, *δ_P_*, *θ_P_*, and *K_R_* were not allowed to vary between individuals). Significant differences are indicated such that “*” indicates p< 0.05,”**” indicates p< 0.01 and “****” indicates p< 0.0001 after Bonferroni test correction. Units of all parameters shown are *d* ^−1^ except for: *k_t_* ([*Ag*]*/d*), *k_b_* ([*A*]^−1^ *d* ^−1^), and *K* ([*Ag*]).

To improve the robustness of our model, we estimated its parameters by fitting the IgG^+^ memory B cell and IgG titer data from the adenovirus based SARS-CoV-2 vaccine study (46), and simultaneously the IgG titer data from an adenovirus-based Ebola study (47). We hypothesized that antigen dynamics parameters may vary by pathogen, and therefore we allowed different values for decay of protein antigen (*δ_Ag_*), and antigen levels for half-maximal proliferation, (*K*) for the two studies. Furthermore, based on preliminary results showing potential differences in long-lived plasma cell generation (*λ*) and decay (*δ_L_*), these parameters were also allowed separate values by pathogen in parameterizing our core model. However, the other parameters were fit to a common population estimate for both studies, with random effects. we found that there were no significant differences between parameters for NHPs receiving two different doses of the Ad-vectored SARS-CoV-2 vaccine. Most parameters also showed no significant difference between the Ad-vectored SARS-CoV-2 vaccine and Ad-vectored Ebola vaccine, with the exception of three parameters: *δ_L_*, *K*, and *δ_Ag_* as shown in **Figure 2**, and confirming our preliminary results. Therefore, in further fits and analyses shown below, we kept most parameter fixed at the values from these initial fits (which we term “core immune parameters”, see **Table 2**), except for: initial reacting B cells (*B*_0_); parameters relating to antigen dynamics (*δ_Ag_*, *k_b_*, *K*, and [for template-based vaccines] *K_R_*); and *δ_L_* and *λ* (as discussed in section, 3.3), which were re-fitted whenever the time frame of the data was long enough to capture this decay rate. In cases where a dataset did not include long enough follow-up data to estimate *λ* and/or *δ_L_*, those parameters were fixed at the values from the training model with the same pathogen (i.e. the Ad-vectored SARS-CoV-2 vaccine or Ad-vectored Ebola vaccine).

**Figure 3:**
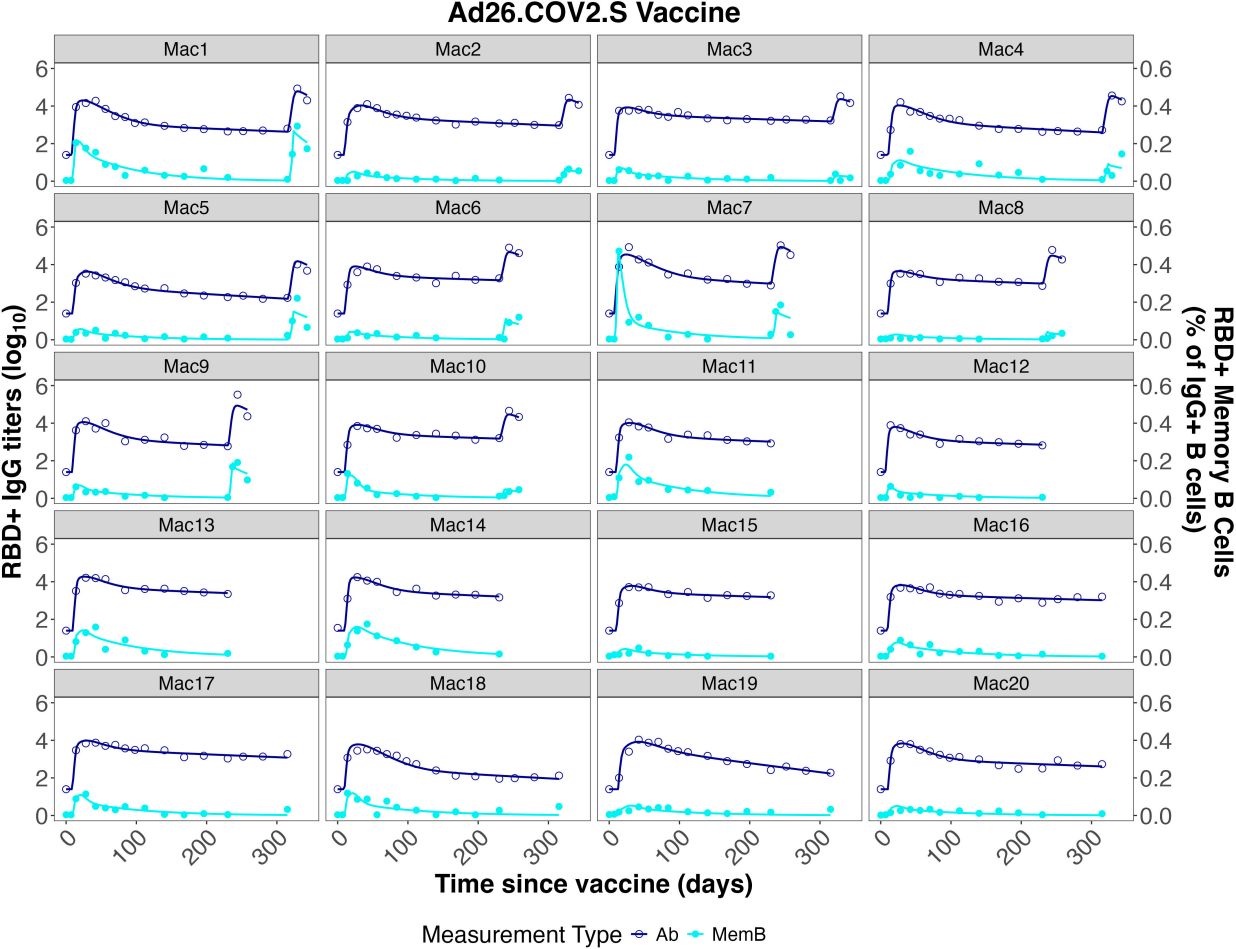
Individual fits to the IgG Ab titer and IgG^+^ memory B cell frequency data from He et al. (46).

**Figure 4:**
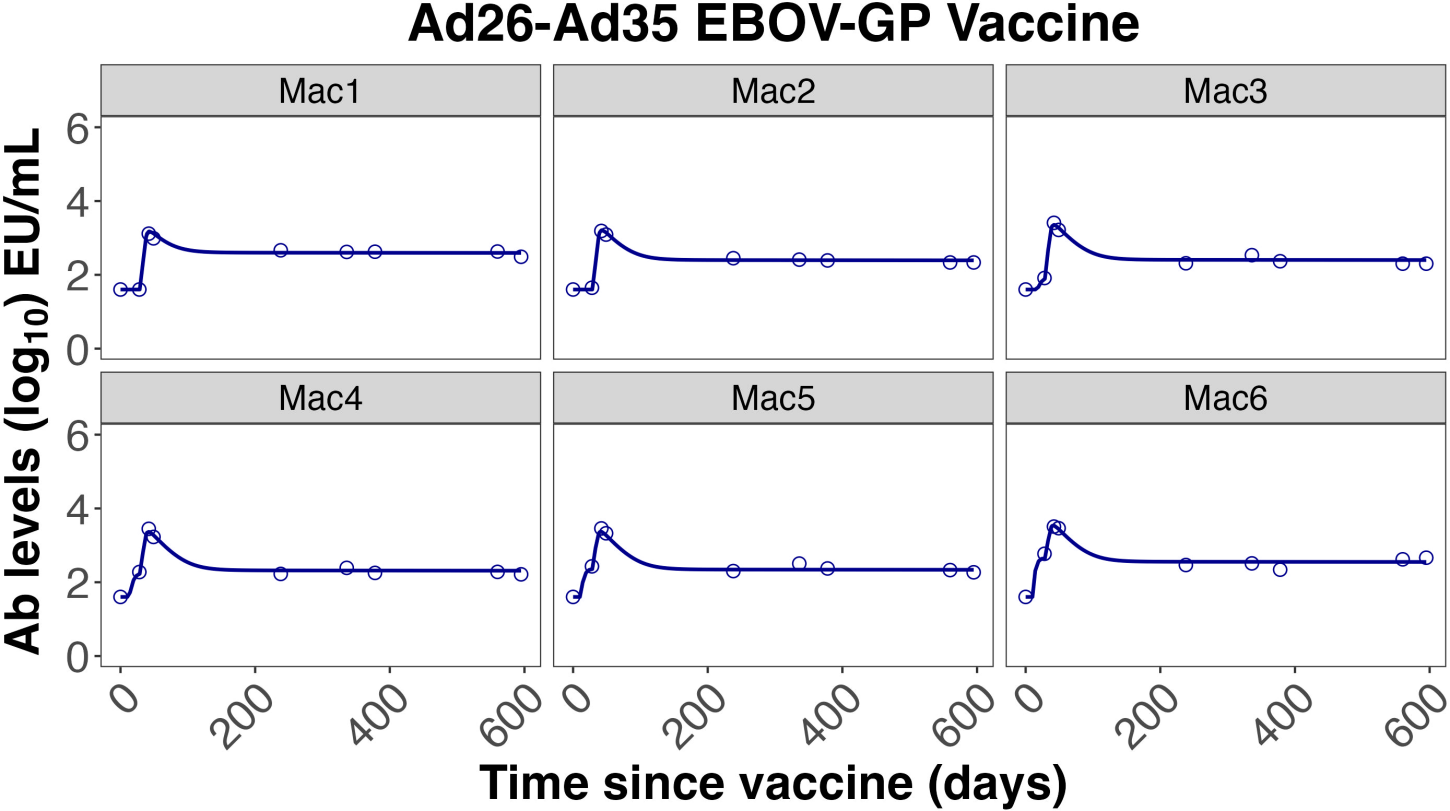
Individual fits to the IgG Ab titer data from Callendret et al. (47).

**Figure 5:**
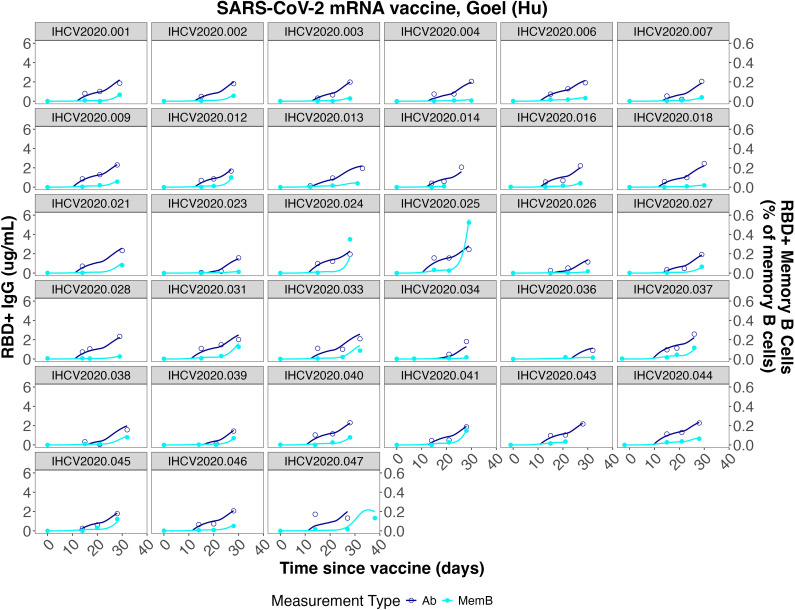
Individual fits to the IgG concentration and IgG^+^ memory B cell frequency data from Goel et al. (7).

As a validation (“out of sample”) test to mitigate over-fitting of the model to these two initial datasets, and to explore the robustness of the parameter estimates, we tested our fits, with core parameters fixed, against a third dataset after mRNA vaccination in humans from (8) (not including individuals with previous SARSCoV-2 infection). We chose this dataset because it contained data both for antibody levels and specific IgG^+^ memory B cells. The core parameter set which performed best overall for both the “construction” and “validation” datasets is shown in **Table 3**, with the scores for the tested parameterizations in **Supplementary Table S3**. While variability in assays used and units reported complicates direct comparison of antibody titers, most parameters in our model are rates with units of only “per time,” and should therefore be applicable across datasets with different parameters. An exception is *k_b_*, which is a second order rate constant with units of $\frac{1}{\text{Time}\times \text{Concentration}}$ , and will depend on whether the Ab unit is concentration or titer. This parameter is re-fit for all datasets. Additionally, because the mRNA vaccination dataset used for validation reported different units for both IgG^+^ antibodies and the B cell frequency, the value for *θ_P_* was re-fit, though the ratios of *θ_L_* and *θ_B_* to *θ_P_* were maintained (1:1 and 0.01:1, respectively). The model described both the memory B cell dynamics and the IgG dynamics from the training and validation datasets very well, as shown in **Figures 3**–**5**.

### 3.3 Sensitivity analysis for antibody peak value and antibody half-life

**Figure 6:**
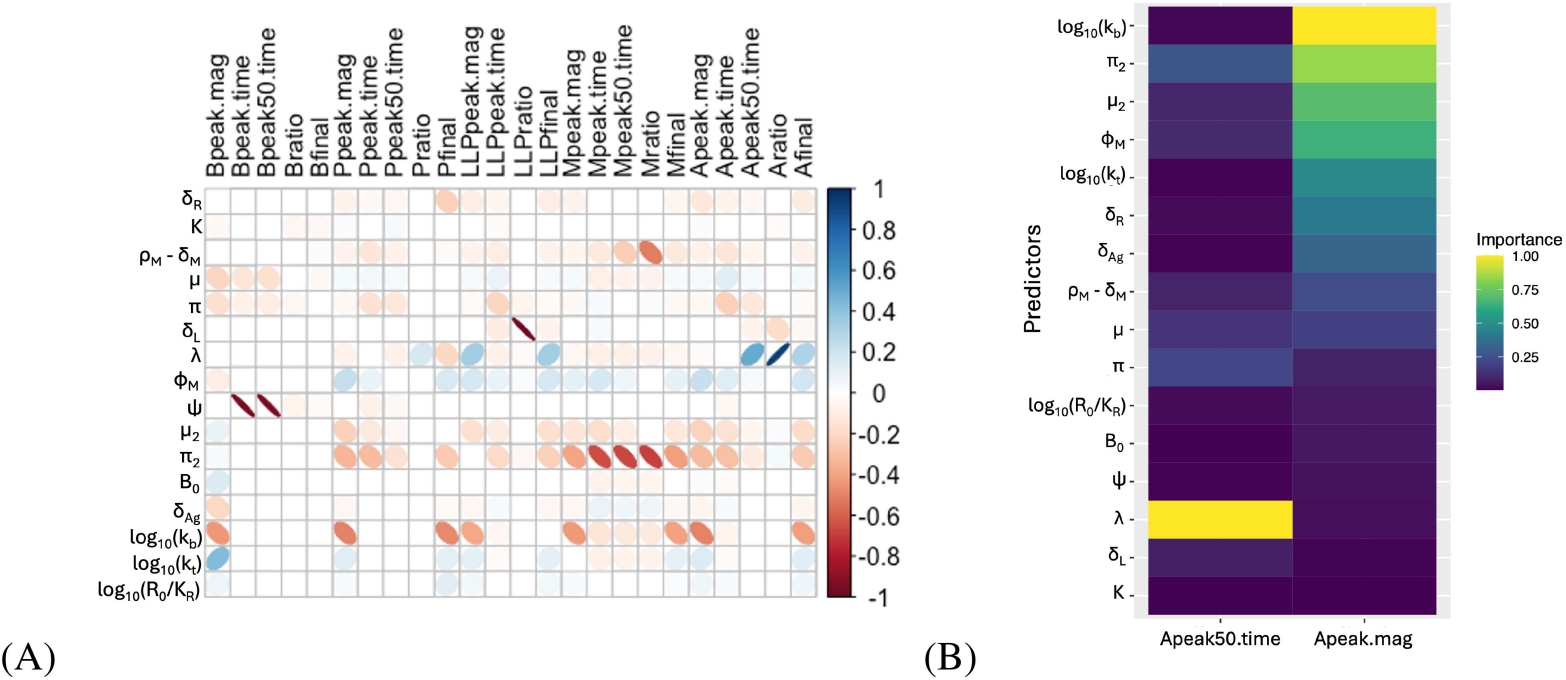
Sensitivity analysis for antibody dynamics. **(A)** Correlations between fit metrics - such as peak timing, peak ratios, etc. - and fit parameters. “Peak” metrics refer to the maximum levels of a species (*P_L_*, *P*, *A*, etc), while “final” metrics refer to the level at the end of the simulated time (315 days), and “ratio” metrics refer to the final value divided by the peak value. Ellipse width correlates with the Bonferronicorrected p-value, with low p values corresponding to very thin ellipses. Empty squares correspond to correlations where p >0.01 after Bonferroni correction. Ellipse color indicates the correlation coefficient according to the scale shown. **(B)** Importance of model parameters, by random forest regression, for prediction of the peak antibody titer and the time to decay to half the peak value.

Since an intended application of our model is to evaluate multiple datasets with minimal re-fitting, we evaluated which parameters were most influential for various model metrics, such as peak antibody titer and time to decay to half of the peak antibody value. We performed 5 × 10^4^ simulations, varying all model parameters with random effects within their fitted distributions from **Table 2**. From these simulations, we calculated metrics describing the simulated curves - peak time, peak magnitude, and time to decay to 50% of the peak value - and calculated the values of those metrics for each major species (GC-experienced activated B cells, short-lived plasma cells (*P*), total memory cells (*M* + *M* ^∗^), long-lived plasma cells (*P_L_*), and antibody). In order to evaluate which parameters are more influential and may need re-fitting based on observed differences in longitudinal Ab titer characteristics between studies, platforms, pathogens, and hosts, we evaluated correlations between these metrics and the simulation parameters, as shown in **Figure 6A**. Unsurprisingly, these correlation results show that the decay rate of long-lived plasma cells has a strong negative correlation with the ratio of final level over peak level for these cells (“LLPratio”). Similarly, the peak time (“Bpeak.time”) and time to decay to 50% of peak (“Bpeak50.time”) for IgG^+^ activated B cells are strongly negatively correlated with the B cell stage transition rate, *ψ*. Perhaps less intuitive is the observation that some parameters defining antigen dynamics, such as *k_t_* or *k_b_*, are also strongly correlated with the magnitude of the response. Furthermore, we made use of a random forest (RF) regression model to evaluate the importance of each parameter in the prediction of antibody peak magnitude (“Apeak.mag”) and the time to decay to 50% of the antibody peak (“Apeak50.time”), which are important parameters in comparing vaccines. **Figure 6B** shows the sensitivity analysis, and predictor parameters for the antibody decay rate and antibody peak value in the RF regression model. Four of the top seven most important predictors of antibody peak value involve antigen dynamics (*e.g., k_b_*, *k_t_*, *δ_R_*, *δ_Ag_*). By contrast, the parameter describing the rate of long-lived plasma cell production, *λ*, is highly predictive of the time to 50% antibody titer decay. The value of *λ* is also strongly correlated with the ratio of the final antibody titer (at 300 days) over the peak antibody titer. Therefore, whenever adequate data were available, the value of *λ* was re-fit for subsequent datasets.

Interestingly, the Ebola protein subunit vaccine in mice produces an earlier Ab peak than seen in other studies which are all performed in non-human primates or humans. We hypothesized that this may have to do with differences in B cell maturation rates for small and short-lived versus large and long-lived hosts (92). Our previous correlation analysis (see **Figure 6A**) suggested that the parameter *ψ* is largely responsible for the timing of the IgG^+^ B cell peak, so we reasoned that re-fitting that parameter for the mouse dataset would allow the model to accommodate earlier B cell peak times, and thereby better capture the Ab peak timing. While the model adequately fits this data without changing core immune parameters, the model fit is improved if *ψ* is re-fit for the mouse model (see **Supplementary Figure S9**). Additional studies are needed comparing the same vaccine platform/pathogen combination across multiple host species to evaluate if the time to the Ab peak differs in different species. However, given the small size and shorter lifespan of mice, it is not unreasonable for the B cell kinetics to vary significantly between mice and NHPs or humans (92). Since this is the only mouse study included in our present analysis we cannot yet verify that re-fitting *ψ* improves the model fit for other mice datasets, and therefore, parsimoniously, we focus the remainder of our analyses on the best fit of this Ebola subunit vaccine in mice with the same value for *ψ* as the datasets in other species.

### 3.4 Testing the model with independent published datasets

**Figure 7:**
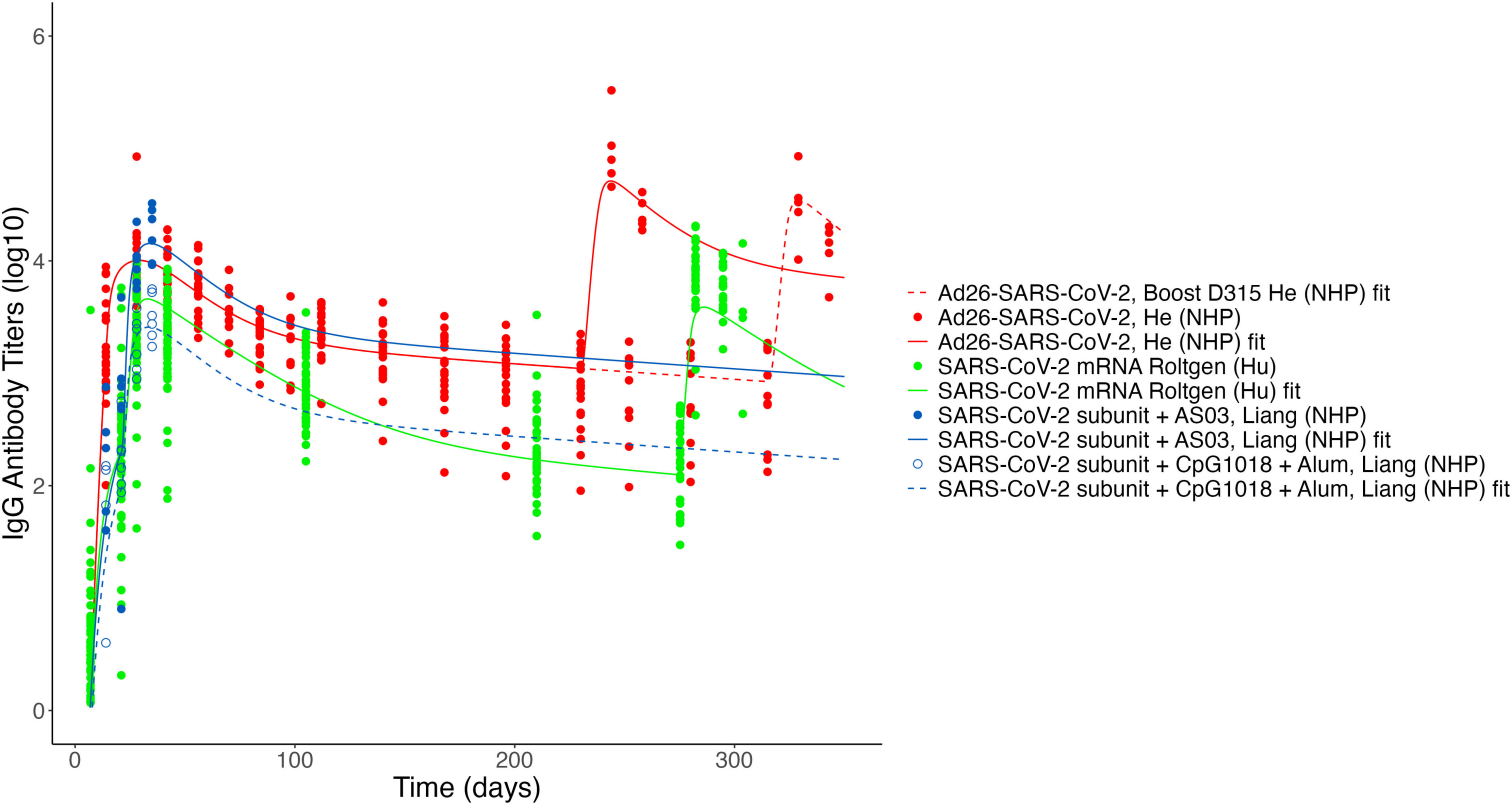
Fits to published Ab titer data from SARS-CoV-2 vaccines. Units are not directly comparable between studies as assays vary. Fits to the SARS-CoV-2 ferritin subunit vaccine (53) and the SARS-CoV-2 mRNA vaccine study by Goel et al. (8) are not shown due to unit differences (AU/mL and *µ*g/mL, respectively).

**Figure 8:**
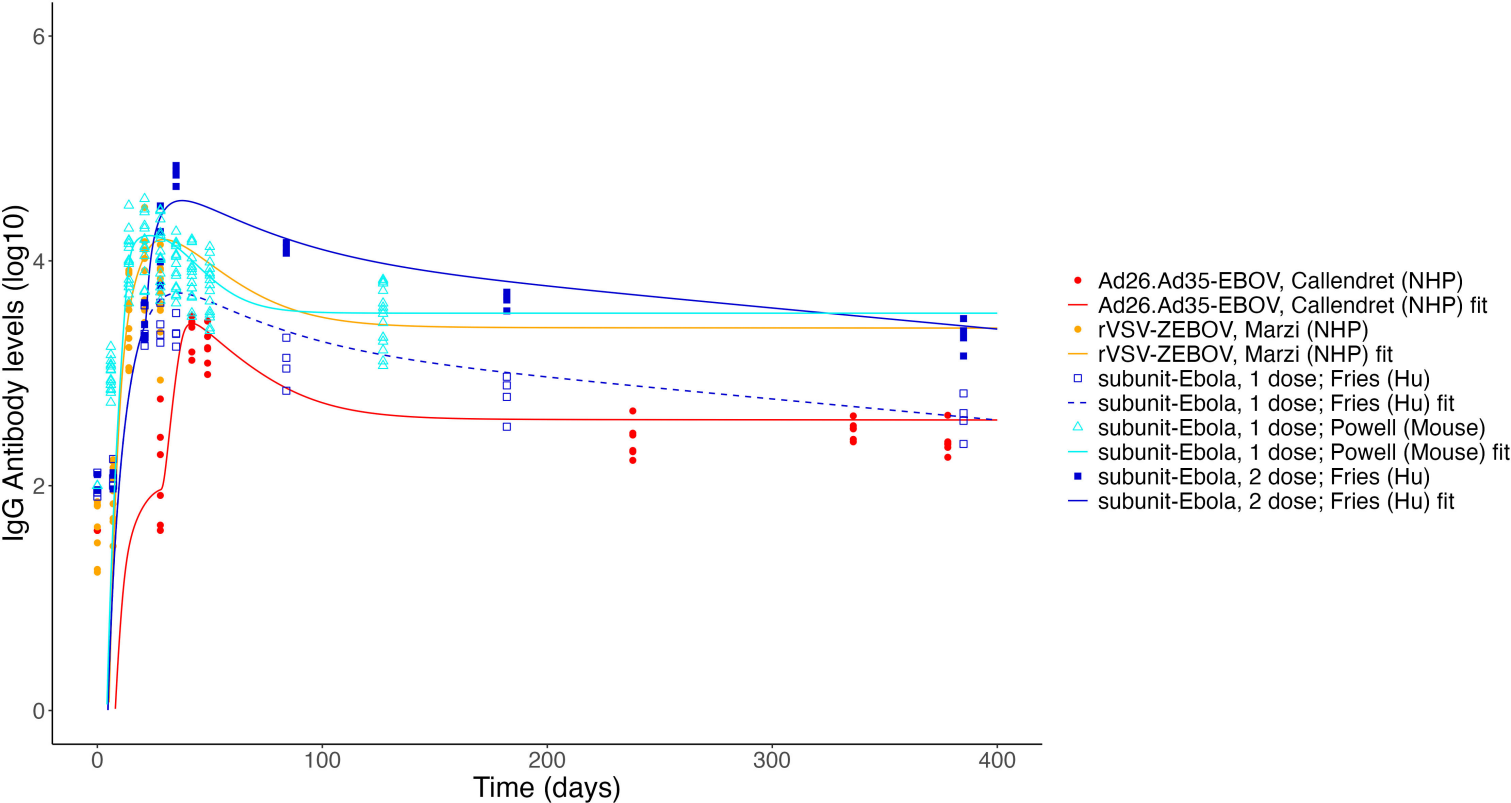
Fits to published Ab titer data from Ebola vaccines. Units are not directly comparable between studies as assays vary.

**Figure 9:**
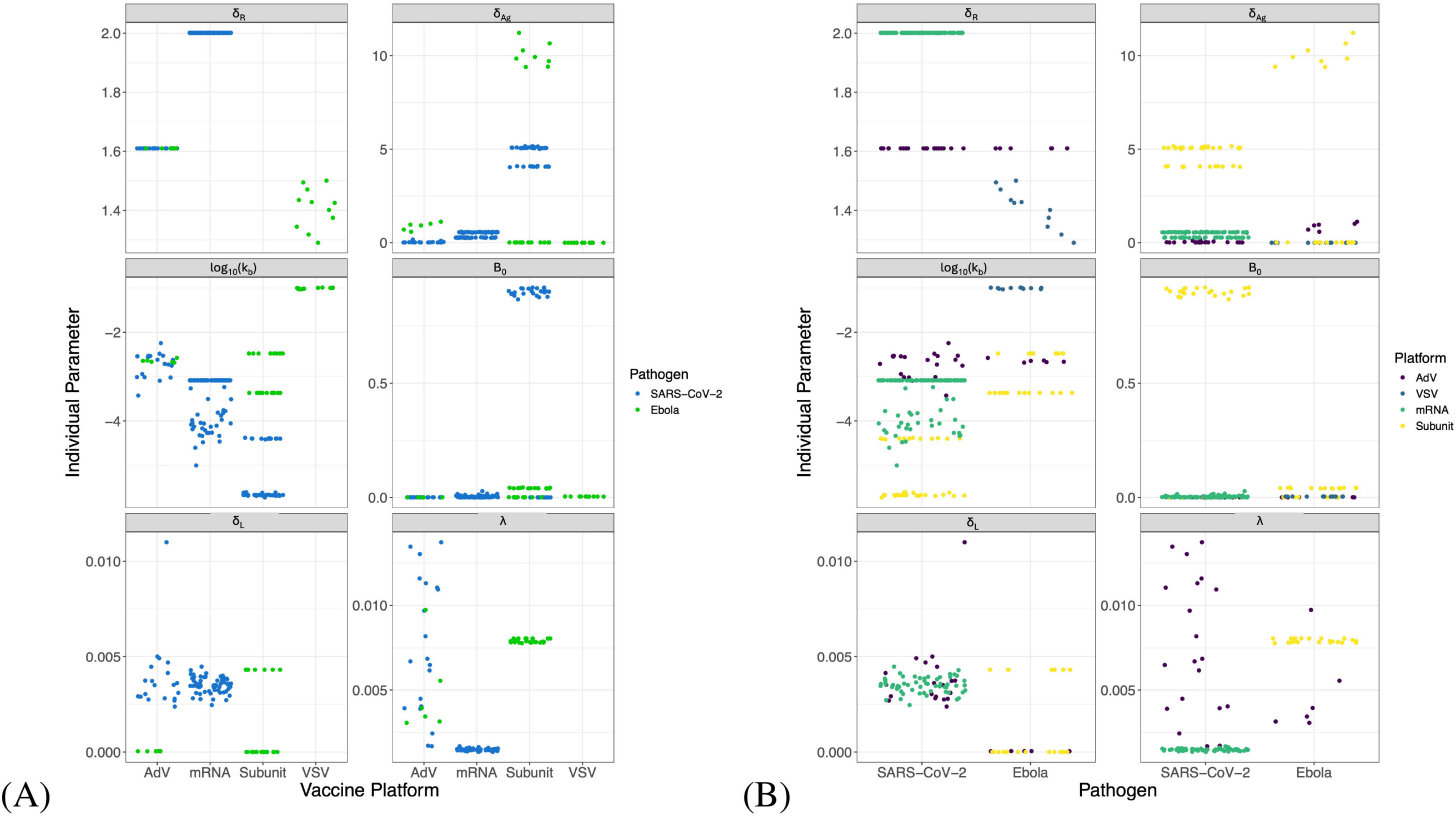
Comparisons of fitted values. Individual parameter values grouped by vaccine platform **(A)** and by pathogen **(B)**. Parameter units shown are *d* ^−1^ except for: *k_b_* ([*A*]^−1^ *d* ^−1^) and *B*_0_ (%, specific B cell frequency). Fitted values for *K_R_* and *K* are not plotted due to differences in units between the studies. Some parameters for specific studies were fitted without random effects, and thus do not show variability.

Considering (1) the consistency of the core model parameters between datasets used, and (2) the importance of antigen dynamics and long-lived plasma cells in predicting key antibody dynamics metrics, we next tested our model on a variety of published datasets reporting longitudinal IgG titers following vaccination against either SARS-CoV-2 or Ebola, fixing “core” parameters and re-fitting only antigen dynamics and *P_L_*-related parameters. The datasets to which we applied the model are described in **Table 1**. Datasets included three SARS-CoV-2 vaccine candidates for two platforms (protein subunit and mRNA), and three Ebola vaccine candidates for two platforms [protein subunit and replicating viral vector (VSV)]. The fits to the Ab levels of individual (or, for Fries et al., mean level across individuals) macaques, mice, or humans are shown in **Supplementary Figures S5**–**S10**. Antibody titer curves generated by the estimated population parameters for each dataset are shown in **Figures 7**, **8**. In all cases, the model described the data with high accuracy while re-fitting only 3–7 parameters for each fit, of which one is the initial cells recruited into the response, *B*_0_, and 2 to 4 relate to Ag dynamics. **Supplementary Table S5** reports the estimated parameters by study and **Supplementary Figure S4** shows a comparison of these parameters, normalized to the maximum value for each parameter. In that figure, we can see that some parameters are very consistent across most studies, such as *B*_0_ or log_10_ *k_B_*. To further compare parameters across platforms and pathogens, we grouped studies according to vaccine platform type (**Figure 9A**) or pathogen (**Figure 9B**). Unfortunately, few platforms were tested on both pathogens. However, where comparable platforms were tested across both pathogens, most fitted parameters showed greater variation between platforms than between pathogens. For example, the AdV-based SARS-CoV-2 vaccine and AdV-based Ebola vaccine we evaluated had similar estimated parameter values for the Ab-mediated antigen clearance rate (*k_b_*), the initial activated B cell fraction (*B*_0_), and the decay rate of presented antigen *δ_Ag_*, while values of those same parameters varied widely within either SARS-CoV-2 vaccines or Ebola vaccines. When considering these parameters, different platforms appear to have differing strengths and weaknesses; the VSV Ebola vaccine had the highest value of *k_b_* of the datasets fitted, while the decay rate of presented antigen (*δ_Ag_*) was generally higher for protein subunit-based vaccines, though the value changed substantially between different subunit formulations. Similarly, long-lived plasma cell generation rates (*λ*) were similar between the two pathogens within the AdV platform. However, as fewer studies included long enough follow-up time points to estimate this parameter and *δ_L_*, the effects of platforms versus pathogens on long-lived plasma cell dynamics are less clear. In general, AdV and subunit vaccines were more efficient at generating long-lived plasma cells (higher *λ*) than the mRNA vaccine for SARS-CoV-2, and the estimated long-lived plasma cell generation rate (*λ*) for the two subunit vaccines for Ebola were higher than most estimates for the AdV-based Ebola vaccine. By contrast, decay rates of long-lived plasma cells generally seemed to be higher for SARS-CoV-2 vaccines than for Ebola vaccines, however the datasets analyzed for subunit-based vaccines for SARS-CoV-2 did not include long-enough follow-up to estimate this parameter.

## 4 Discussion

In fitting a mathematical model of vaccine-induced antibody dynamics to both IgG titers and memory B cell data, we can gain potential insights into the immune response to vaccination. We showed that differing Ab dynamics between multiple platforms and pathogens could be described with our model by re-fitting only a small subset of the model parameters - those having to do with the dynamics of presented antigen and with the generation and longevity of long-term immune cells. The ability to fix most core immune dynamics parameters supports our hypothesis that many underlying immune processes, such as transition rates between types of immune cells involved in the acute response to vaccination, are consistent between vaccine platforms and antigens. Our consensus mathematical model suggests that plasma cells are produced far more rapidly from activated memory cells than from other activated IgG^+^ B cells, in agreement with experimental data (58). It is interesting to note that the estimated parameter values for the relative production of memory cells versus plasma cells in our model indicate a higher production rate of memory cells. This result was consistent across all of the best-scoring model parameterizations for the “construction” and the “validation” datasets. While we might expect a more even split between plasma cells and memory cells (97), the skewness towards development of memory cells over plasma cells is consistent with previous reports which have found that robust long-term immunity is better with “repetitive antigens”, such as mumps and measles (98). In our model, repeated exposure to an antigen, *e.g.* by boosting or potentially activation of early memory cells, would improve production of long-lived plasma cells by allowing memory cell-derived short-lived plasma cells another chance to become long-lived plasma cells.

Using our mechanistic model calibrated with both longitudinal specific IgG titers and memory B cell frequencies, we are able to accurately capture the dynamics of the IgG titers in response to vaccines for two different pathogens, with four unique platform types, and three host species. In addition to facilitating fits to more sparse datasets by fixing the core immune parameters, use of a common model structure facilitates comparison between different datasets. We noted that, for most parameters, The results of our fits suggest that SARS-CoV-2 mRNA vaccines may be less efficient at generating long-lived plasma cells than the Ad26-SARS-CoV-2 vaccine (**Figure 9A**). This result is consistent with reports that long-lived plasma cells do not establish in the bone marrow following mRNA SARS-CoV-2 vaccination (45). Indeed, mRNA vaccines appear to be the least efficient at generating long-lived plasma cells. However, long-lived plasma cell decay rates also seem to differ between SARS-CoV-2 vaccines and Ebola vaccines (**Figure 9B**), with SARS-CoV-2 vaccines generally having lower rates of long-lived plasma cell generation and higher decay rates of long-lived plasma cells. These results suggest some antigen-specific effects on long-lived plasma cell generation and maintenance. However, all SARS-CoV-2 vaccines with long-enough follow up to re-fit *δ_L_* were template-based vaccines (AdV and mRNA); longer-term follow up with other platforms, such as protein subunit vaccines may lead to better estimates of long-lived plasma cell decay rates. Efficiency of long-lived plasma cell generation is difficult to compare between the two pathogens due to a seemingly strong influence from the vaccine platform. Additional studies, with similar protocols testing the same vaccine platform across multiple antigens and hosts, could help to determine whether such differences are the result of platform or host species differences, or antigen choice. Our results also showed *k_b_* values were highest for the VSV-vector vaccine, suggesting that antibody-mediated clearance of vaccine antigen may play a more significant role in the B-cell-mediated immune response to VSV-vectored vaccines than other vaccines.

We were able to fit varied datasets modifying mostly the antigen-related parameters, suggesting that much of the difference in immune response between vaccine platforms is related to antigen dynamics and antigen presentation. Our model is, thus, well-positioned to investigate effects of prolonging antigen duration or promoting better antigen presentation on enhancing immune responses in future work. Our results also indicate that experimental studies comparing antigen dynamics between different vaccine platforms may provide insight into whether differences in immune responses are induced by the differences in antigen format, adjuvants, or administration methods between vaccine platforms. Finally, we also note high variability in some parameters associated with antigen dynamics, such as *δ_Ag_*, *k_b_*, and *B*_0_, particularly within the subset of protein subunit-based vaccine platforms. This is reasonable due to the very heterogeneous nature of this vaccine platform; studies grouped into this platform vary in adjuvants, antigen structure (e.g. different fusion proteins), size (e.g. trimers, multimers), and administration route (intramuscular versus subcutaneous). Targeted studies systematically exploring these variables may help to elucidate their effects on antigen dynamics and the downstream immune response.

Our modeling approach has some limitations. Clearly the model proposed is a simplification of the complex processes involved in generating an immune response to a vaccine. For example, we do not explicitly model the details of the germinal center reaction (see (99) for a recent detailed model of this process). We also do not model CD4^+^ T-cell help, which is very important especially in B-cell memory formation. These choices are based on not having enough data to parameterize a more complex model; for instance, there is little data on help provided by CD4^+^ T cells in the vaccination studies we used here; or on choosing to keep a level of simplification commensurate with the data we want to describe. Thus, we do not model the details of the affinity maturation process but they are implicit in our model in a simplified way, such as the *B*_1_ to *B*_8_ cascade for the GC reaction. Another limitation is that the datasets are typically not consistent in terms of assays used, units used, and time of follow-up. In this regard, we note that rate parameters should be independent of the units of measurement, and most of our parameters are such rates. In addition, our mixed-effect approach borrows information across the different datasets, mitigating issues of different times of follow-up. However, better data, more frequent with longer follow-up and a larger dynamic range would help modeling efforts.

In conclusion, our work has shown that immune responses to vaccination can be successfully mapped onto a common consensus mathematical model structure. Furthermore, our work suggests that many immune dynamics parameters can be held constant between pathogens, hosts, and vaccine platforms. Additional, controlled comparisons between vaccine antigens, vaccine platforms, or hosts may enable identification of platform-, antigen-, or host-specific parameters which could then be pieced together to predict the success of untested combinations of antigen/platform.

## Data Availability

The original contributions presented in the study are included in the article/**Supplementary Material**, further inquiries can be directed to the corresponding author/s.
