# Supplementary material for: A consensus mathematical model of vaccine-induced antibody dynamics for multiple vaccine platforms and pathogens
Source: Front Immunol. 2025 Jun 26;16:1596518. doi: 10.3389/fimmu.2025.1596518 (PMC12241011; doi:10.3389/fimmu.2025.1596518)
Supplement: Supplementary file 1 [file DataSheet1.pdf]

frontiers

Supplementary Material

1 ADDITIONAL INFORMATION ON ANTIGEN TEMPLATE DYNAMICS FITS

As described in the main text, we include in our humoral immune response model the dynamics of antigen presentation. For some vaccine platforms, we were able to find data on either the antigen template (*e.g.*, mRNA) or the antigen protein. For these cases, we fitted the antigen dynamics part of the model independently to the data from those studies. For adenovirus-based vaccines, we used the rAd5 template levels over time, as reported by Quinn *et al.* [1] to approximate the rAd26 dynamics (in our SARS-CoV-2 vaccine data set), as the rAd5 had the most measurements above the detection limit. Additionally, in later applications of the rAd-vectored vaccine, a booster dose using rAd5 was added, suggesting that the response is comparable between vectors [2]. These data were fit to Eq. 10 in the main text using Monolix. Adenovirus vaccines deliver antigen as DNA, which is then quickly transcribed into mRNA. The initial rise in mRNA transcripts was not detectable in the experiments, suggesting mRNA levels peak quickly before 8 hours (the time of the first measurement). Therefore, we make a quasi-steady state assumption that mRNA dynamics are fast and are reflective of the underlying template dynamics. Thus, absent any replication of an underlying template (*e.g.,* replicating viral vectors or self-amplifying RNA), mRNA dynamics is dominated by template decay from an initial peak value which we assume is proportional to the template administered. Thus, for the fit to Eq. 10, we set r and T_{off} to 0. Dynamics for the mRNA template in standard mRNA vaccines was set by simultaneoulsy fitting Equations 9 and 10 in the main text to mRNA H-score and EGFP positivity data from [3] after mRNA-LNP vaccination in NHPs. As with the adenovirus vaccines, r and T_{off} were set at 0, since the mRNA is not replicating. Since the antigen, as well as mRNA and protein level units differed significantly from those used in the other vaccination studies, only the fitted template decay rate, δ_R, was used as a fixed value for subsequent mRNA vaccine fits. VSV dynamics was estimated by fitting Eq. 9 in the main text to peripheral rVSV transcript levels reported by Dahlke *et al.* [4] after rVSV-EBOV vaccination in humans. Fits to the template data are shown in Figures S1 through S3. Parameter values from the fitted template data which were fixed in other fits are shown in Table S1.

2 ADDITIONAL INFORMATION ON ALTERNATIVE MODELS TESTED

We tested various alternatives of the model structure presented in the main text, while retaining the same antigen structure (R and Ag). For details, see Table S2.

A model (S0) without long-lived plasma cells is unable to capture the two-phase antibody decay, with antibody instead decaying to very low levels as short-lived plasma cells are depleted. Models without an activated memory cell stage, which allow faster proliferation and differentiation to plasma or resting memory cells (models S1 and S2), are unable to capture the magnitude of the booster responses unless Ag persists for long enough to allow a new wave of GC B cells to develop from B1 to B8. However, this contradicts experimental reports which have found that additional IgG+ cells after a boost are generated primarily from IgG+ memory and not from newly class-switched responses [5]. We also tested several models (S3 to S8 in Table S2) with different number of stages of B_i cells, with and without antigen removal by antibody, and different forms of the proliferation rate of activated memory cells (M^*).

As mentioned in the main text, each of these models was fitted starting from at least 20 different initial guesses, resulting in slightly different values of BICc. To fit the validation dataset, we used not only the

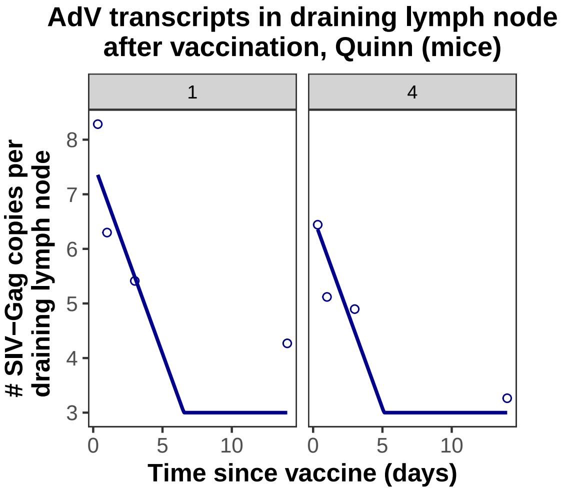

Figure S1. Fits to AdV transcript in draining lymph node, data from [1].

very best model in the construction datasets, but also several models with the best BICc scores (Table S3). In each of these cases, we kept the values of the core model parameters fixed (*i.e.,* these were not fitted to the validation dataset) and estimated the others. We then chose the model that fitted both the construction and validation dataset best. Interestingly, when fitting just the construction datasets, model S3 was best, but adding the validation dataset showed that model S7 was more flexible. The only difference between these two model structures is that the proliferation of activated memory cells (M^*) has a saturation term (S7) instead of a linear one (S3). Thus, the model structure presented in the main text is that of S7. However, Monolix was unable to estimate standard errors for the parameters of the top scoring model parameterization, S7-1. We therefore selected the next best overall model parameterization: model S7-3. The population parameter values are very similar between S7-3 and S7-1, since these only differ in the initial guesses for the parameters.

3 SUPPLEMENTARY TABLES AND FIGURES

Table S1. Fitted antigen dynamics parameters used in other fits

Parameter [s.e.]	AdV	VSV	mRNA	unit
r	0	2.08 [0.7]	0	d^{-1}
T_{off}	0	1.68 [0.18]	0	d
δ_R	1.61 [0.61]	1.4 [0.24]	2.0 [0.42]	d^{-1}
$log_{10}(x)$ (scaling)	1.41 [0.48]	-5.98 [0.45]	0.12 [0.08]	unitless

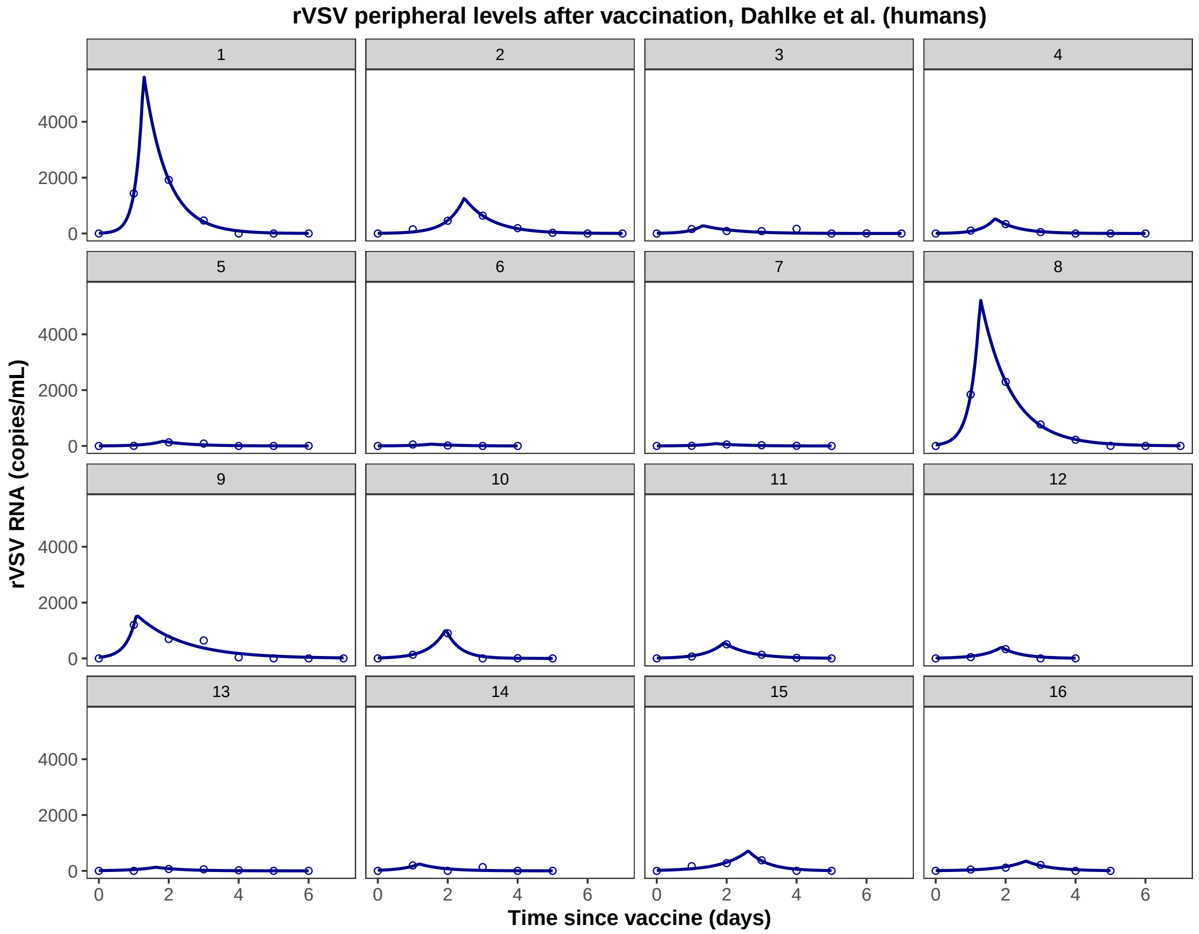

Figure S2. Fits to rVSV transcript data from [4].

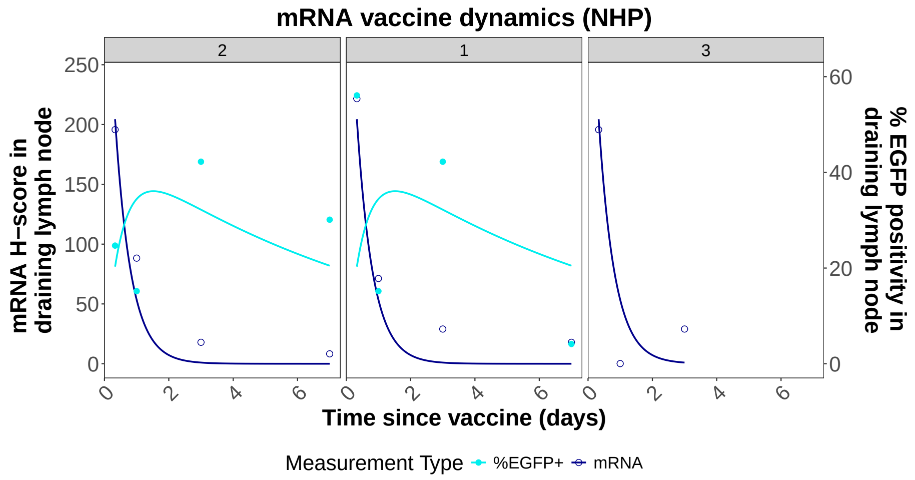

Figure S3. Fits to mRNA and EGFP positivity data from mRNA vaccine study [3].

Table S2. Best likelihood scores of model structure variations

Model	Model description	-2LL	BICc
S0	No LLPs, activated M return to B_8	179.78	343.92
S1	With LLPs, activated M return to GC (B_8)	-1192.91	-996.48
S2	With LLPs, activated M become M*, μ_2=μ, π_2=π	-1193.91	-997.47
S3	eight B cell stages (pre-GC / GC), with LLPs, activated M become M*, M* proliferate at max prolif. rate	-1318.46	-1096.15
S4	S3 with two B cell stages, no Ab-Ag binding (pre-GC / GC)	-805.56	-631.55
S5	S3 with two B cell stages, no Ab-Ag binding (pre-GC / GC)	-1081.84	-907.82
S6	S3 with two B cell stages (pre-GC / GC) and Ab-Ag binding	-1276.76	-1054.35
S7	S3 with M* proliferate at saturating rate	-1321.59	-1089.51
S8	S7 with μ=π	-1312.86	-1090.46

Table S3. Log-likelihood and BICc scores against training and validation for top model parameterizations.

	Construction		Validation		Overall
Model	-2LL	BICc	-2LL	BICc	-2LL
S3-1	-1318.46	-1096.15	-630.29	-568.58	-1948.75
S8-1	-1312.86	-1090.46	-687.88	-626.16	-2000.74
S7-1	-1321.59	-1089.51	-693.93	-632.21	-2015.52
S8-2	-1311.68	-1089.27	-689.92	-628.21	-2001.6
S7-2	-1321.18	-1089.1	-685.46	-623.74	-2006.64
S8-3	-1309.96	-1087.56	-689.69	-627.97	-1999.65
S7-3	-1314.68	-1082.6	-694.77	-633.05	-2009.45

Table S4. Dose information on published datasets

Author	Pathogen	Vaccine platform	Species	Dose	Booster days	Reference
He et al.	SARS-CoV-2	Ad Vector	rhesus macaques	1E11, 2E10 vp	230 or 315 days	[6]
Callendret et al.	EBOV	Ad Vector	cynomolgus macaques	8E10 vp (total)	28 days	[7]
Goel et al.	SARS-CoV-2	mRNA	humans	assume 30 ug	about 21 days	[8]
Roltgen et al.	SARS-CoV-2	mRNA	humans	30 ug	21 days, about 9 months	[9]
Joyce et al.	SARS-CoV-2	protein subunit	rhesus macaques	5 or 50 ug	28 days	[10]
Liang et al.	SARS-CoV-2	protein subunit	rhesus macaques	30 ug	21 days	[11]
Marzi et al.	EBOV	VSV vector	cynomolgus macaques	1E07 PFU		[12]
Fries et al.	EBOV	protein subunit	humans	50 ug	day 21	[13]
Powell et al.	EBOV	protein subunit	C57BL/6 mice	5 ug		[14]

Table S5. Fitted population parameters

Data Source	B_0 (%)	$log_{10}(K_R)$ log_{10}([R])	$log_{10}(K)$ log_{10}([Ag])	$log_{10}(k_b)$ $log_{10}(\frac{1}{[A]*d})$	δ_{Ag} (d^{-1})	λ (d^{-1})	δ_L (d^{-1})
He [6]	5.5E-04 [4.6E-03]	3.49 [0.67]	4.99 [1.14]	-2.74 [0.18]	0.014 [0.055]	0.0058 [0.0013]	0.0032 [0.00061]
Goel [8]	4.8E-03 [6.5E-04]	7.55 [0.073]	-0.8 [0.068]	-4.02 [0.69]	0.27 [0.022]		
Roltgen [9]	1.1E-03 [4.2E-04]	4.77 [0.23]	0.9 [0.083]	-3.08 [0.11]	0.57 [0.021]	0.0015 [5.5E-04]	0.0036 [0.0023]
Liang [11] (CpG+Alum)	1.5E-04 [1.2E-03]		-0.51 [0.83], 0.034 [0.051]	4.4 [3.46]	4.07 [3.46]		
Joyce [10]	0.9 [1.33]		0.79 [2.76]	-5.67 [2.37]	5.07 [272.98]		
Callendret [7]	5.3E-04 [2.5E-02]	3.49 [0.67]	6.05 [1.91]	-2.7 [0.18]	0.9 [0.52]	0.0049 [0.0015]	3.8E-05 [NaN]
Marzi [12]	4.1E-03 [NaN]	-4.55 [NaN]	4.72 [0.31]	-1.01 [0.24]	1.3E-08 [NaN]		
Fries [13]	1.1E-03 [2.9E-04]		-4.12 [0.37]	-2.48 [0.077]	9.97 [1.0]	0.0081 [0.0016]	0.0043 [0.0005]
Powell [14] (Ferritin 24-mer) (trimer)	4.1E-02 [5.9E-02]		2.2 [0.83], 2.19 [0.87], 2.9 [1.33]	-3.37 [0.5]	0.013 [NaN]	0.0078 [0.0025]	1.7E-08 [0.23]

NOTE: In some cases, it was not possible to evaluate the uncertainty in the estimation of a parameter, and its S.E. is specified as NaN. Note that NaN does not affect the maximum likelihood estimation.

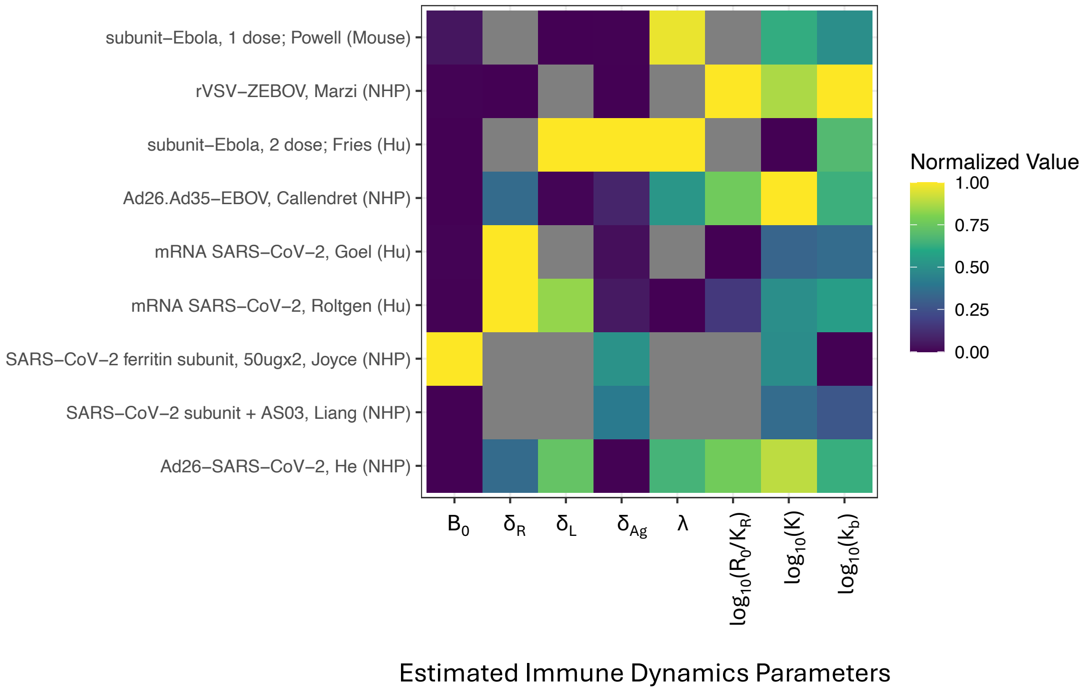

Figure S4. Heatmap showing values of varying parameters for fits to each dataset, normalized to the min and max fitted value of each parameter ($(x - \min)/(\max - \min)$).

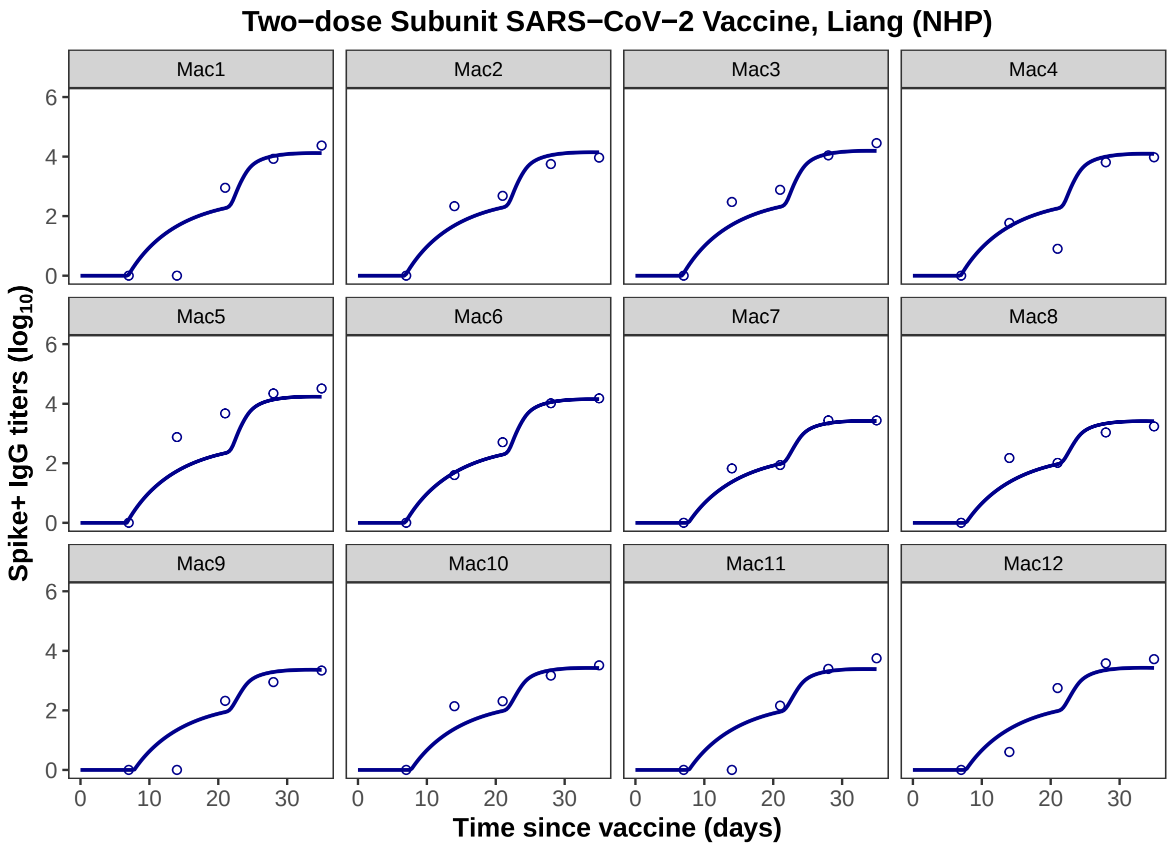

Figure S5. Individual fits to the IgG Ab titer data from Liang et al. SARS-CoV-2 vaccination study in non-human primates (NHP).

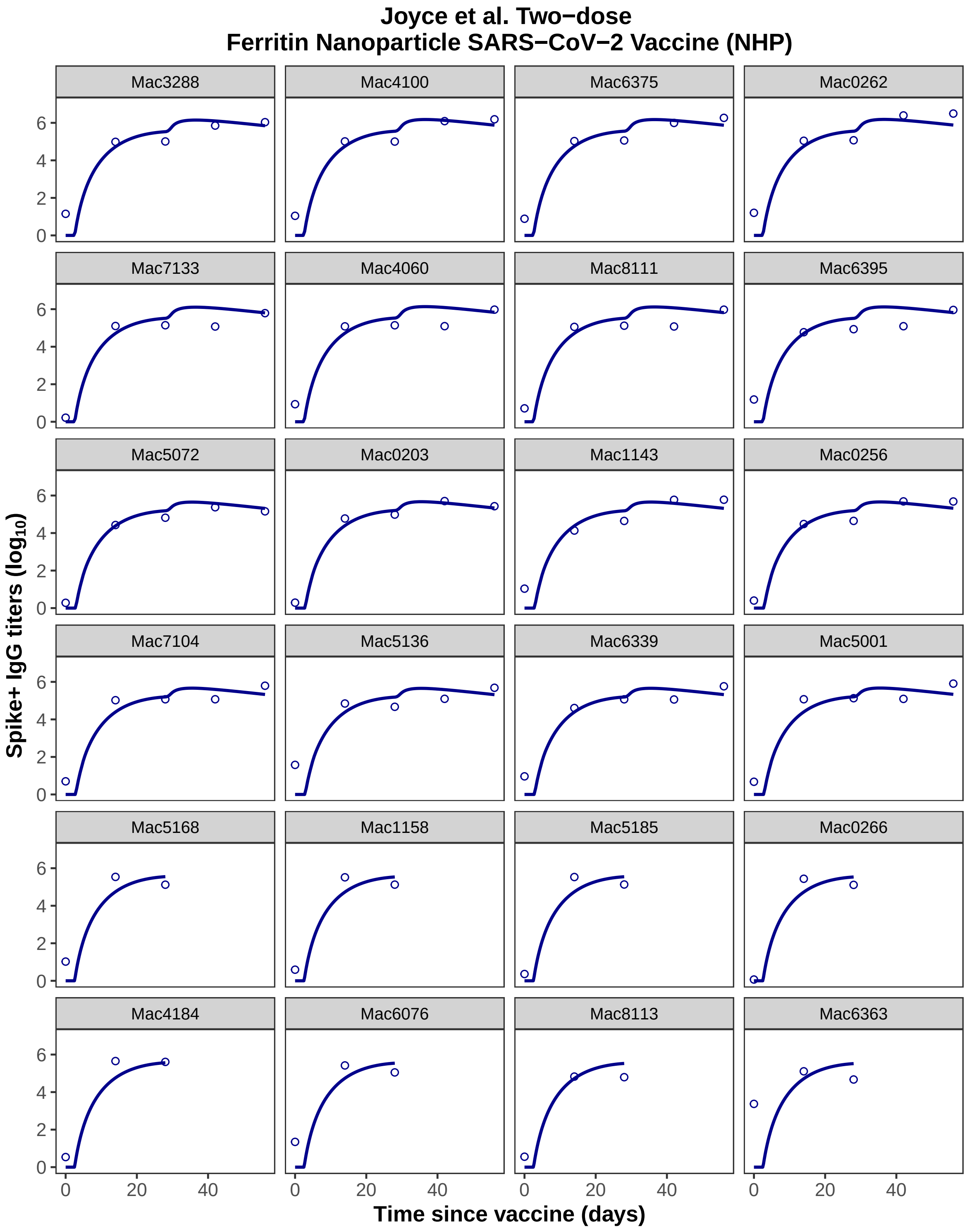

Figure S6. Individual fits to the IgG Ab titer data from Joyce et al. SARS-CoV-2 vaccination study in NHP.

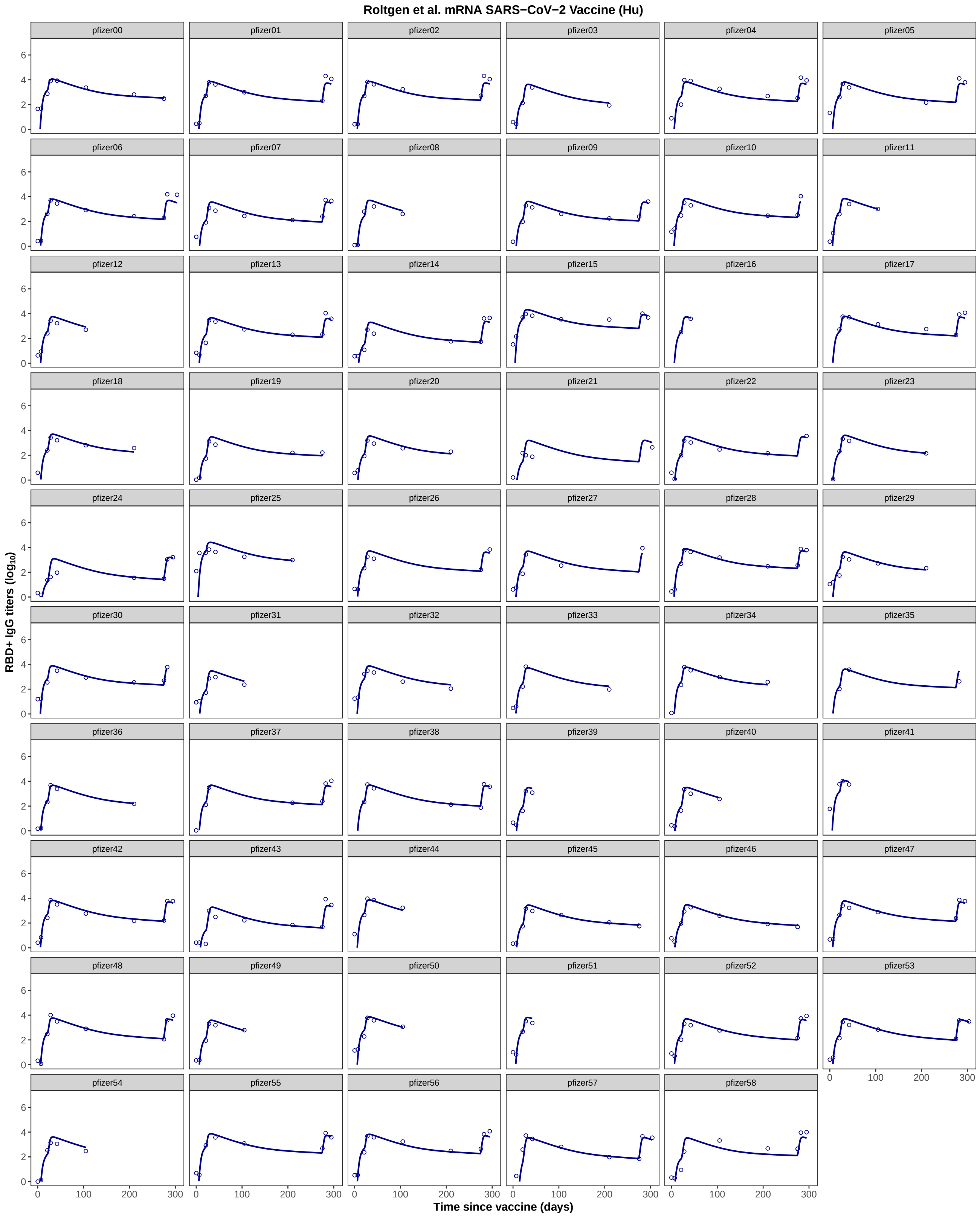

Figure S7. Individual fits to the IgG Ab titer data from Roltgen et al. SARS-CoV-2 vaccination study in humans.

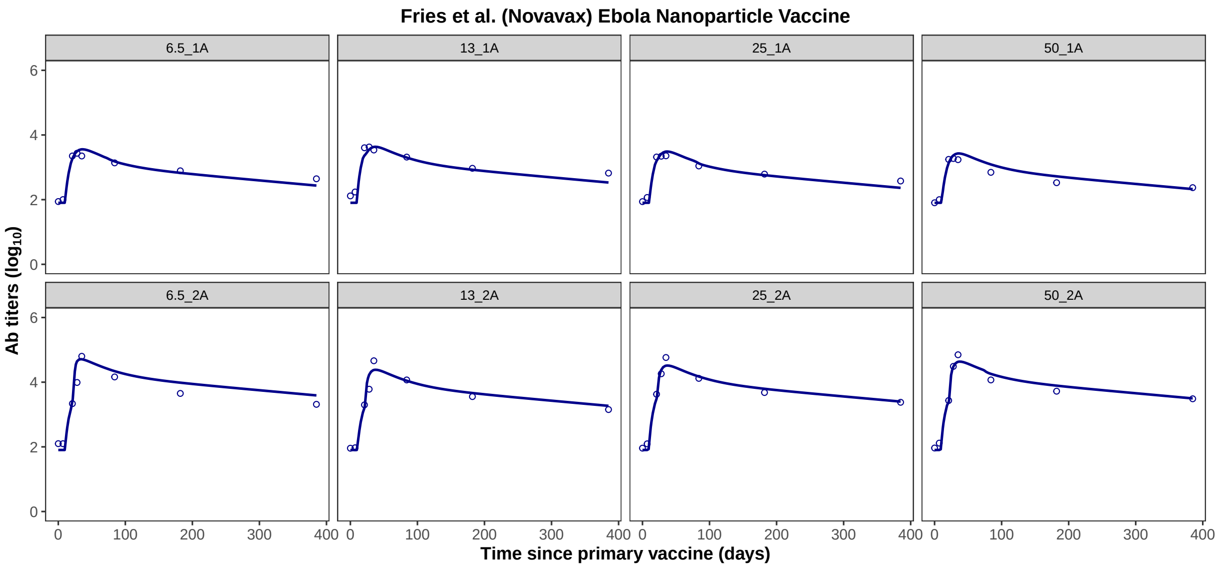

Figure S8. Individual fits to the IgG Ab titer data from Fries et al. Ebola vaccination study in humans

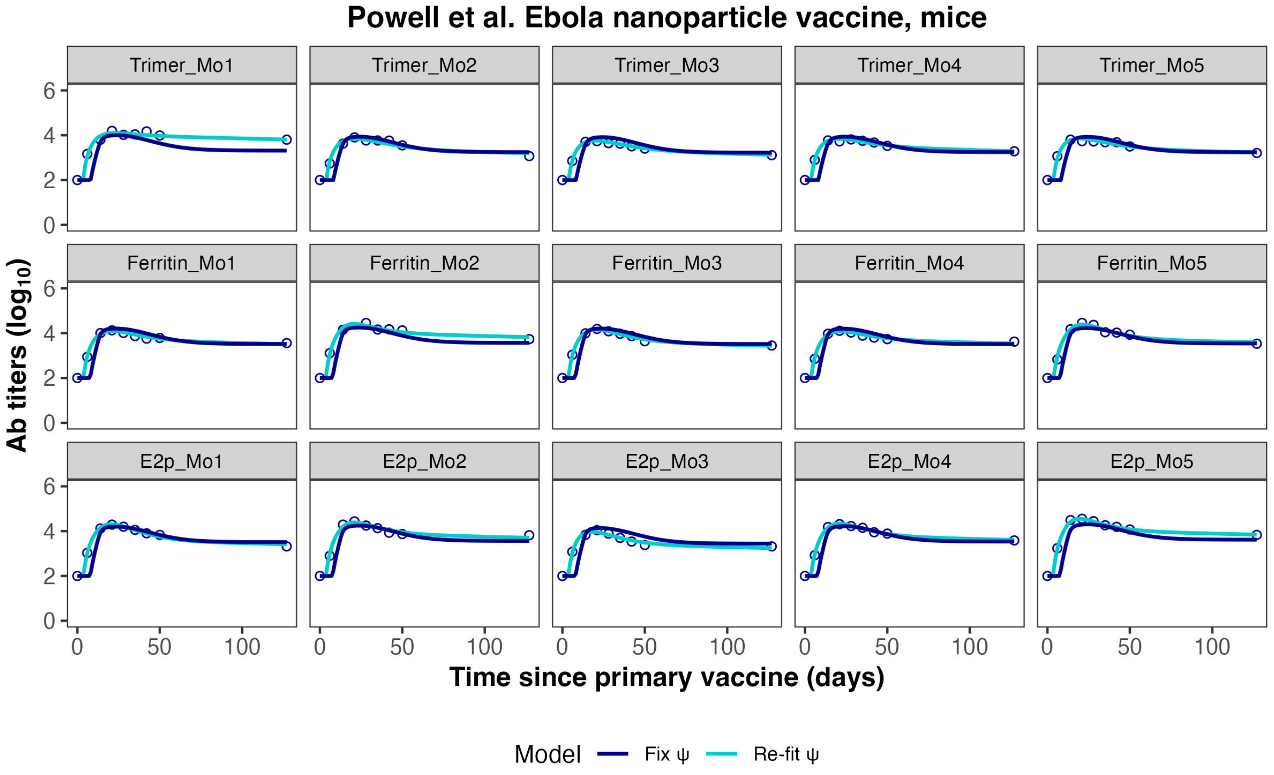

Figure S9. Individual fits to the IgG Ab titer data from Powell et al. Ebola vaccination study in mice. Dark blue line indicates the fit using the same value of ψ used in fits for NHP and humans. Cyan line indicates the fit when ψ is re-fit for this mouse dataset.

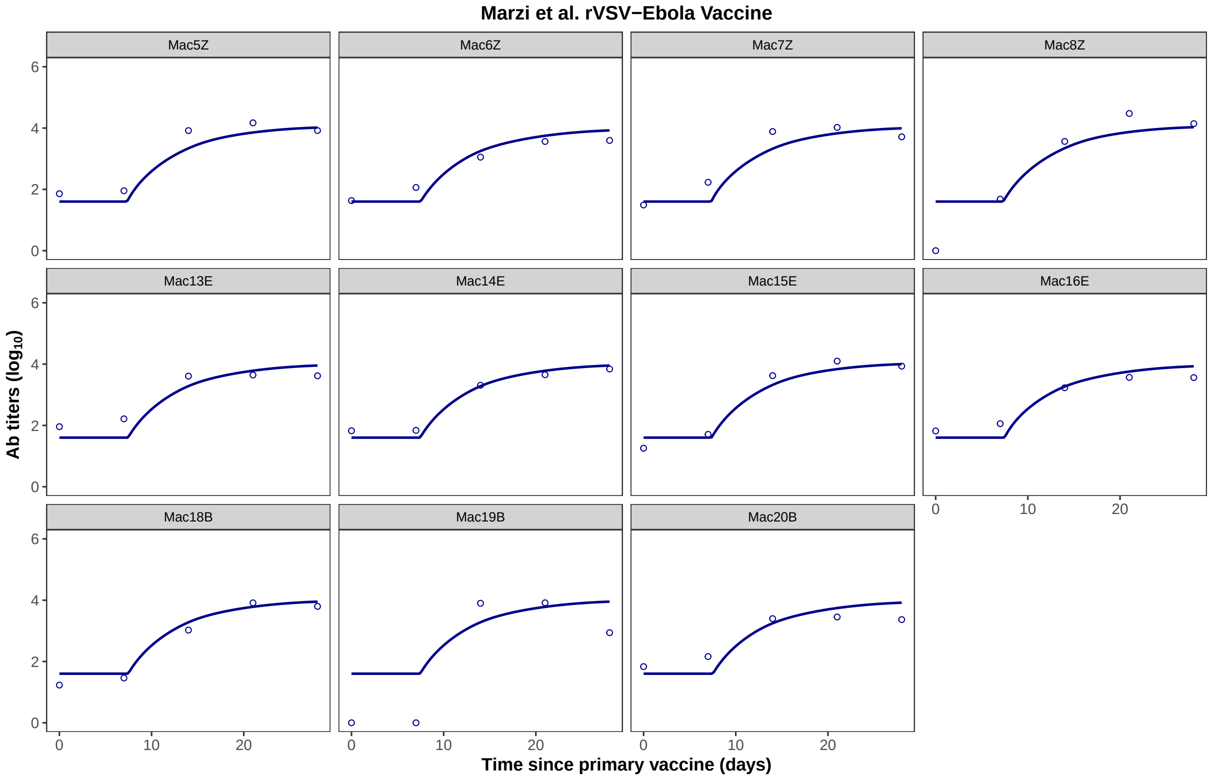

Figure S10. Individual fits to the IgG Ab titer data from Marzi et al. Ebola vaccination study in NHP.

REFERENCES

[1] Kylie M Quinn, Daniel E Zak, Andreia Costa, Ayako Yamamoto, Kathrin Kastenmuller, Brenna J Hill, Geoffrey M Lynn, Patricia A Darrah, Ross WB Lindsay, Lingshu Wang, et al. Antigen expression determines adenoviral vaccine potency independent of ifn and sting signaling. *J Clin Invest*, 125(3):1129–1146, 2015.

[2] Maria G Byazrova, Ekaterina A Astakhova, Aygul R Minnegalieva, Maria M Sukhova, Artem A Mikhailov, Alexey G Prilipov, Andrey A Gorchakov, and Alexander V Filatov. Anti-ad26 humoral immunity does not compromise sars-cov-2 neutralizing antibody responses following gam-covid-vac booster vaccination. *npj Vaccines*, 7(1):145, 2022.

[3] Kimberly J Hassett, Ivana Liric Rajlic, Kapil Bahl, Rebecca White, Kristen Cowens, Eric Jacquinet, and Kristine E Burke. mrna vaccine trafficking and resulting protein expression after intramuscular administration. *Molecular Therapy-Nucleic Acids*, 35(1), 2024.

[4] Christine Dahlke, Rahel Kasonta, Sebastian Lunemann, Verena Krähling, Madeleine E Zinser, Nadine Biedenkopf, Sarah K Fehling, My L Ly, Anne Rechtien, Hans C Stubbe, et al. Dose-dependent t-cell dynamics and cytokine cascade following rvsv-zebov immunization. *EBioMedicine*, 19:107–118, 2017.

[5] Kathryn A Pape, Justin J Taylor, Robert W Maul, Patricia J Gearhart, and Marc K Jenkins. Different b cell populations mediate early and late memory during an endogenous immune response. *Science*, 331(6021):1203–1207, 2011.

[6] Xuan He, Malika Aid, Abishek Chandrashekar, Jingyou Yu, Katherine McMahan, Frank Wegmann, Catherine Jacob-Dolan, Jenny S Maron, Caroline Atyeo, Huahua Wan, et al. A homologous or variant booster vaccine after ad26. cov2. s immunization enhances sars-cov-2–specific immune responses in rhesus macaques. *Sci Transl Med*, 14(638):eabm4996, 2022.

[7] Benoit Callendret, Jort Vellinga, Kerstin Wunderlich, Ariane Rodriguez, Robin Steigerwald, Ulrike Dirmeier, Cedric Cheminay, Ariane Volkmann, Trevor Brasel, Ricardo Carrion, et al. A prophylactic multivalent vaccine against different filovirus species is immunogenic and provides protection from lethal infections with ebolavirus and marburgvirus species in non-human primates. *PLOS One*, 13(2):e0192312, 2018.

[8] Rishi R Goel, Sokratis A Apostolidis, Mark M Painter, Divij Mathew, Ajinkya Pattekar, Oliva Kuthuru, Sigrid Gouma, Philip Hicks, Wenzhao Meng, Aaron M Rosenfeld, et al. Distinct antibody and memory b cell responses in sars-cov-2 naïve and recovered individuals after mrna vaccination. *Science Immunol*, 6(58):eabi6950, 2021.

[9] Katharina Röltgen, Sandra CA Nielsen, Oscar Silva, Sheren F Younes, Maxim Zaslavsky, Cristina Costales, Fan Yang, Oliver F Wirz, Daniel Solis, Ramona A Hoh, et al. Immune imprinting, breadth of variant recognition, and germinal center response in human sars-cov-2 infection and vaccination. *Cell*, 185(6):1025–1040, 2022.

[10] M Gordon Joyce, Hannah AD King, Ines Elakhal-Naouar, Aslaa Ahmed, Kristina K Peachman, Camila Macedo Cincotta, Caroline Subra, Rita E Chen, Paul V Thomas, Wei-Hung Chen, et al. A sars-cov-2 ferritin nanoparticle vaccine elicits protective immune responses in nonhuman primates. *Sci Transl Med*, 14(632):eabi5735, 2021.

[11] Joshua G Liang, Danmei Su, Tian-Zhang Song, Yilan Zeng, Weijin Huang, Jinhua Wu, Rong Xu, Peiwen Luo, Xiaofang Yang, Xiaodong Zhang, et al. S-trimer, a covid-19 subunit vaccine candidate, induces protective immunity in nonhuman primates. *Nat Commun*, 12(1):1346, 2021.

[12] Andrea Marzi, Flora Engelmann, Friederike Feldmann, Kristen Haberthur, W Lesley Shupert, Douglas Brining, Dana P Scott, Thomas W Geisbert, Yoshihiro Kawaoka, Michael G Katze, et al. Antibodies

are necessary for rvsv/zebov-gp–mediated protection against lethal ebola virus challenge in nonhuman primates. *Proc Natl Acad Sci USA*, 110(5):1893–1898, 2013.

[13]Louis Fries, Iksung Cho, Verena Krähling, Sarah K Fehling, Thomas Strecker, Stephan Becker, Jay W Hooper, Steven A Kwilas, Sapeckshita Agrawal, Judy Wen, et al. Randomized, blinded, dose-ranging trial of an ebola virus glycoprotein nanoparticle vaccine with matrix-m adjuvant in healthy adults. *J Inf Dis*, 222(4):572–582, 2020.

[14]Abigail E Powell, Duo Xu, Gillie A Roth, Kaiming Zhang, Wah Chiu, Eric A Appel, and Peter S Kim. Multimerization of ebola gpδmucin on protein nanoparticle vaccines has minimal effect on elicitation of neutralizing antibodies. *Front Immunol*, 13:942897, 2022.
